# Targeting metastasis-initiating cancer stem cells in gastric cancer with leukaemia inhibitory factor

**DOI:** 10.1038/s41420-024-01839-1

**Published:** 2024-03-07

**Authors:** Lornella Seeneevassen, Anissa Zaafour, Elodie Sifré, Coralie Genevois, Tra Ly Nguyen, Yasmine Pobiedonoscew, Alban Giese, Jérôme Guignard, Camille Tiffon, Benoit Rousseau, Anne-Aurélie Raymond, Geneviève Belleannée, Hélène Boeuf, Caroline Gronnier, Océane C. B. Martin, Julie Giraud, Philippe Lehours, Pierre Dubus, Christine Varon

**Affiliations:** 1 INSERM U1312, Bordeaux Institute of Oncology, University of Bordeaux, 33076 Bordeaux, France; 2 VIVOPTIC TBM-Core, University Bordeaux, CNRS UAR3427 INSERM US005, 33076 Bordeaux, France; 3 Animal Facility, University of Bordeaux, 33076 Bordeaux, France; 4 Oncoprot TBM-Core, University of Bordeaux, CNRS UAR3427 INSERM US005, 33076 Bordeaux, France; 5 CHU Bordeaux, F-33076 Bordeaux, France; 6 Department of Histology and Pathology, CHU Bordeaux, F-33000 Bordeaux, France; 7 INSERM U1026, Tissue Bioengineering, University of Bordeaux, Bordeaux, France; 8 Department of Digestive Surgery, Haut-Lévêque Hospital, F-33000 Bordeaux, France; 9 Centre National de Référence des Campylobacters et Helicobacters, Pellegrin Hospital, Bordeaux, 33076 France

**Keywords:** Extracellular signalling molecules, Cancer stem cells, Gastric cancer

## Abstract

Gastric cancer’s (GC) bad prognosis is usually associated with metastatic spread. Invasive cancer stem cells (CSC) are considered to be the seed of GC metastasis and not all CSCs are able to initiate metastasis. Targeting these aggressive metastasis-initiating CSC (MIC) is thus vital. Leukaemia inhibitory factor (LIF) is hereby used to target Hippo pathway oncogenic members, found to be induced in GC and associated with CSC features. LIF-treated GC cell lines, patient-derived xenograft (PDX) cells and/or CSC tumourspheres underwent transcriptomics, laser microdissection-associated proteomics, 2D and 3D invasion assays and in vivo xenograft in mice blood circulation. LIFR expression was analysed on tissue microarrays from GC patients and in silico from public databases. LIF-treated cells, especially CSC, presented decreased epithelial to mesenchymal transition (EMT) phenotype and invasion capacity in vitro, and lower metastasis initiation ability in vivo. These effects involved both the Hippo and Jak/Stat pathways. Finally, GC’s high LIFR expression was associated with better clinical outcomes in patients. LIF treatment could thus represent a targeted anti-CSC strategy to fight against metastatic GC, and LIFR detection in primary tumours could constitute a potential new prognosis marker in this disease.

## Introduction

GC is a deadly disease accounting for ~768,793 deaths worldwide in 2020, making it the 4th leading cause of cancer-related death [1]. GC therapy comprises surgery with additional adjuvant or neo-adjuvant chemo- and radio-therapies and the number of relapses remains high. Surgical resection which remains the only curative therapeutic option is possible in case of early tumours but GC is commonly a late-diagnosed disease with 80% of patients detected at metastatic state and with a 5-year survival rate of <5% [2, 3]. It is thus crucial to find appropriate diagnostic tools for earlier detection of GC as well as strategies for better-targeted therapy of disseminated cancers.

The most aggressive component of heterogeneous gastric tumours is the rare population of cancer stem cells (CSC) which carry specific self-renewal and chemo-/radio-resistant properties [4–7], allowing them to initiate tumours and cause recurrence. In the metastatic process, part of these CSC corresponding to metastasis-initiating CSC (MIC), is able to acquire invasive characteristics, evade the primary tumour, disseminate, and colonise distant organs to initiate metastases [8, 9]. We have recently identified CD44v3+ cells as MIC in gastric tumours, associated with bad prognosis in GC [8] and which could constitute an interesting therapeutical target for GC metastatic disease. CD44v3+ cells are a subpopulation of gastric CD44+ CSC and our previous work has demonstrated an enrichment of the Hippo pathway oncogenic members Yes-associated protein (YAP) and transcriptional co-activator with PDZ-binding motif (TAZ) in GC and especially in gastric CSC [10–12]. YAP and TAZ are well-described for their role in invasion and metastasis and their targeting could affect GC MIC.

We have demonstrated that activating the Hippo kinase core with leukaemia inhibitory factor (LIF) decreases tumorigenic properties of gastric CSC [4]. Nevertheless, LIF/LIF receptor (LIFR) signalling was never tested in the gastric metastatic context despite its promising anti-metastatic effects in hepatocellular carcinoma [13], clear cell renal carcinoma [14] and breast cancer [15].

LIF–LIF receptor (LIFR) canonical pathway is mediated by JAK/STAT activation, causing STAT3 phosphorylation, dimerisation, and nuclear translocation, resulting in the transcription of target genes involved in cell survival and tumorigenesis [16]. Other cell signalisation processes have been identified downstream of LIF-LIFR, which could explain its pleiotropy [16–23]. Nonetheless, LIF-LIFR-dependent anti-metastatic properties in breast cancer implicated the Hippo pathway activation [15].

The aim of this study was to decipher LIF’s potential as anti-metastatic therapy in GC and the underlying signalling mechanisms. We have shown that LIF decreased invasive properties of GC cell lines and patient-derived xenograft (PDX) cells and more especially gastric CSC in vitro through activation of Hippo kinases and repression of YAP/TAZ oncogenic signalling. LIF’s anti-invasive function involves the repression of CD44v3 expression and of the epithelial-to-mesenchymal transition (EMT) programme in CSC, important in the GC metastatic process. LIF-treated cells were also less keen to form metastasis compared to non-treated cells when injected into mice’ blood circuits. Finally, LIFR’s high expression in gastric tumours seems to be protective since it is correlated to better prognosis of GC patients despite them having CD44v3+ MIC-rich or mesenchymal ZEB1+ tumours.

## Results

### LIF decreases EMT- and invasion-related genes’ expression in GC cells

**Fig. 1:**
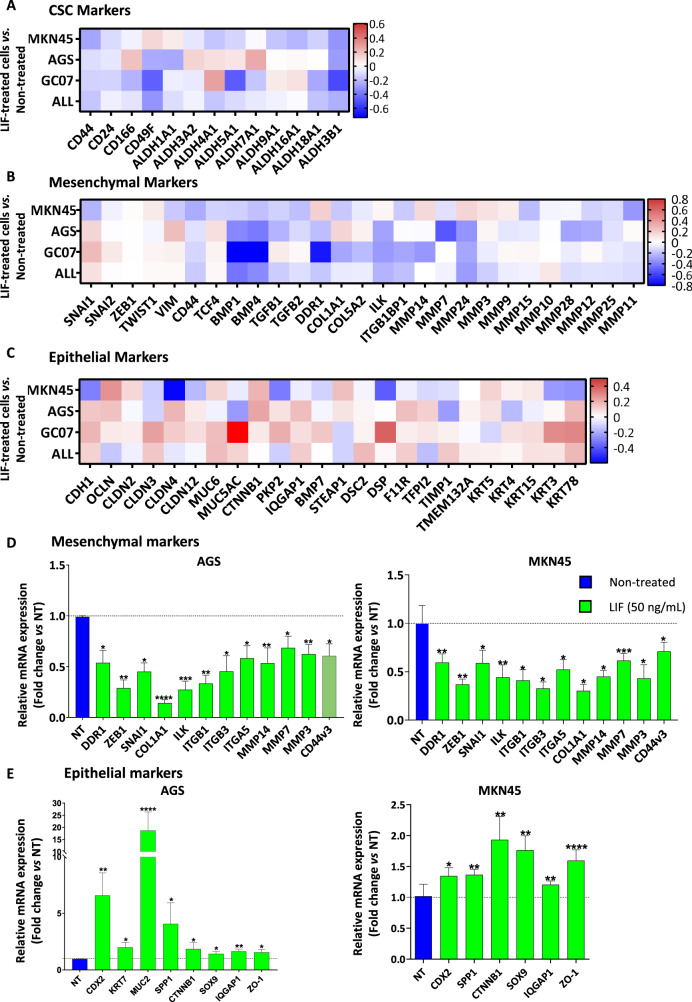
Leukaemia inhibitory factor presents an EMT-inhibiting signature in GC cells. Relative LIF-treated cells gene expression profiles showing **A** CSC markers, **B** mesenchymal markers and **C** epithelial markers expression in MKN45 and AGS GC cell lines and GC07 PDX cells. Agilent microarray transcriptomic analysis was carried out on LIF-treated cells compared to non-treated cells. The fourth row represents the mean expression fold change in all the cells analysed. Relative mRNA expressions of **D** mesenchymal and **E** epithelial markers, assessed by RT-qPCR, after treatment of AGS and MKN45 cells with (green) or without (blue) LIF. LIF treatments (50 ng/mL) were carried out for 48 h. Values represent fold change vs. non-treated cells, 3 < *n* < 4. *p < 0.05, **p < 0.005, ***p < 0.0005 and ****p < 0.0001 vs. untreated controls with ANOVA statistical analyses.

Transcriptomic analysis of LIF-treated GC cells MKN45, AGS and GC07 PDX compared to non-treated ones showed that LIF decreased gastric CSC markers expression (Fig. 1A). A decrease in Hippo oncogenic signature was also noted as shown preceding [4], through decrease in TAZ, TEAD and their main target genes expression but increase in Hippo partners, known to work with Hippo kinases to inhibit YAP/TAZ-TEAD oncogenic activity (Supplementary Fig. S1A) [24]. Importantly, LIF decreased the expression of mesenchymal markers and increased epithelial ones (Fig. 1B-C), showing its possible role in the EMT process known to trigger invasion and metastasis in cancer [25, 26].

In this respect, LIF’s effect on epithelial and mesenchymal marker expression was assessed by RT-qPCR. Results showed an increase in epithelial markers and a decrease in mesenchymal markers, including *ZEB1* and *SNAI1* EMT-transcription factors (EMT-TF) (Fig. 1D, E), reflecting a decrease in the EMT process in GC cells. In addition, several matrix metalloproteases (MMP), linked to the capacity of cells to invade extracellular matrix (ECM), also decreased showing a possible inhibitory effect of LIF on GC cell invasion ability. LIF also decreased CD44v3 mRNA expression (Fig. 1D) revealing that it not only affects gastric CSC population as described previously [4] but can also target CD44v3+ MIC, involved in GC chemoresistance, invasion and metastasis and is related to poor prognosis in patients [8]. To evaluate LIFR implication in the observed effects, doxycycline-inducible LIFR knocked down (LIFR-KD) GC cells were established (Supplementary Fig. S2A–C). LIF treatment was no longer able to decrease the expression of EMT-related genes nor CD44v3 CSC marker in LIFR-KD GC cells (Supplementary Fig. S2D, E), suggesting that LIF signals through LIFR to induce these effects.

### LIF decreases EMT-TF nuclear expression

**Fig. 2:**
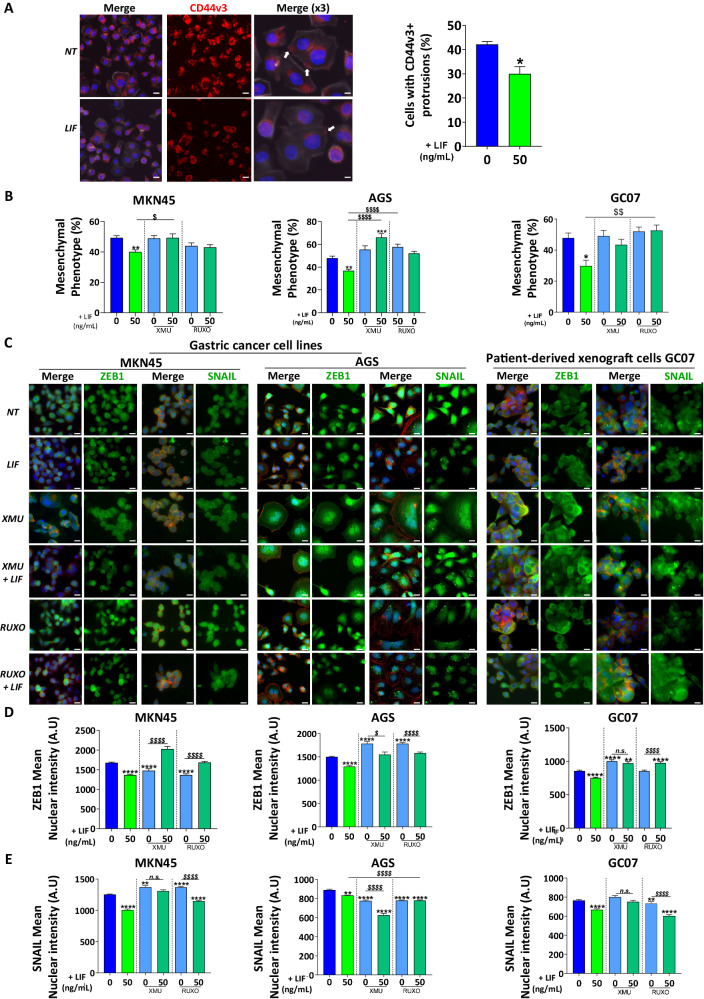
Leukaemia inhibitory factor decreases EMT-associated transcription factors nuclear expression and EMT phenotype of GC cells. **A** Representative immunofluorescence images of AGS GC cell lines stained with anti-CD44v3 antibody (red). Cells with CD44v3-positive cell protrusions (white arrows) were quantified. All cells were marked with phalloidin (grey) and DAPI (blue). Scale bars 10 µm. **B** Mesenchymal phenotype quantification of MKN45 and AGS GC cell lines and GC07 PDX cells. Quantifications were done on phalloidin-stained cells and values represent the percentage of cells with mesenchymal phenotype of *n* = 3 experiments ± SEM. **C** Representative immunofluorescence images of MKN45 and AGS GC cell lines and GC07 GC PDX cells stained with anti-ZEB1 or anti-SNAIL antibodies (green). All cells were marked with phalloidin (red) and DAPI (blue). Scale bars 10 µm. **D** and **E** Relative quantification of relative nuclear expression of ZEB1 or SNAIL in cells. Values represent mean nuclear intensity ± S.E.M., 3 < *n* < 4. All cells were treated with 50 ng/mL LIF (green) and/or 0.5 µM XMU-MP-1 (XMU) (emerald green) and 1 µM Ruxolitinib (emerald green) for 48 h. Inhibitors were added 30 min before LIF stimulation. *p < 0.05, **p < 0.005, ***p < 0.0005 and ****p < 0.0001 vs. untreated controls and $*p* < 0.05, $$*p* < 0.005 and $$$$*p* < 0.0001 vs. the conditions indicated by the bars, all with ANOVA statistical analyses.

LIF’s effect on CD44v3 expression was further assessed at the protein level. CD44v3 was localised at cell protrusions which might be related to its pro-invasive function (Fig. 2A). LIF treatment decreased the number of cells having CD44v3 protrusions (Fig. 2A) suggesting an anti-invasive effect of LIF on GC cells.

LIF’s effect on EMT phenotype was analysed by counting the number of GC cells carrying an elongated protrusion-rich phenotype [7, 11] after treatment (Fig. 2B). Indeed, LIF decreased EMT phenotype in GC cells confirming the decrease observed in EMT-related gene expression (Fig. 1D–E). Moreover, to evaluate the implication of the Hippo and JAK/STAT signalling pathways in LIF-observed effects, cells were treated with XMU-MP-1 (XMU) or Ruxolitinib inhibiting Hippo kinase MST1/2 upstream LATS1/2 and JAK kinases, respectively. LIF’s anti-EMT effect was blocked in the presence of both inhibitors (Fig. 2B) showing both Hippo and JAK/STAT pathways implication.

Since EMT-TF ZEB1 and SNAIL activity is related to their sub-cellular localisation, their expression was evaluated in the nucleus where they control the expression of EMT-related genes. LIF significantly decreased ZEB1 and SNAIL nuclear expression (Fig. 2B–D), which was correlated with the reduced expression of EMT-associated genes as well as in EMT phenotype observed in LIF-treated GC cells (Figs. 1B–E, 2B). These effects were demonstrated in GC cell lines MKN45 and AGS, and also in PDX-derived cells GC07 and were blocked upon LIFR-KD in these cells (Supplementary Fig. S3A–C), demonstrating the role of LIFR in the observed effects.

In the presence of XMU, LIF was no longer able to decrease ZEB1 and SNAIL nuclear expression in MKN45 and GC07 cells (Fig. 2B–D), showing a possible role of the Hippo pathway in LIF effects. On the other hand, Ruxolitinib-mediated JAK/STAT pathway inhibition reduced ZEB1 nuclear translocation, and not that of SNAIL showing that both pathways were implicated in EMT decrease through ZEB1 but only the Hippo pathway controls SNAIL in this context. An exception was noted for the AGS cell line where inhibitors completely blocked LIF repression of nuclear ZEB1 (Fig. 2B–D). Moreover, in AGS cells, only the JAK/STAT pathway was involved in the LIF-related decrease of nuclear SNAIL.

LIF thus targets GC CD44v3+ CSC harbouring mesenchymal phenotype and affects EMT phenotype and EMT-TF’s activity and this, through LIFR and either Hippo and/or JAK/STAT or alternative pathways [16], depending on the model.

### LIF presents anti-EMT and anti-invasive properties in GC cells

**Fig. 3:**
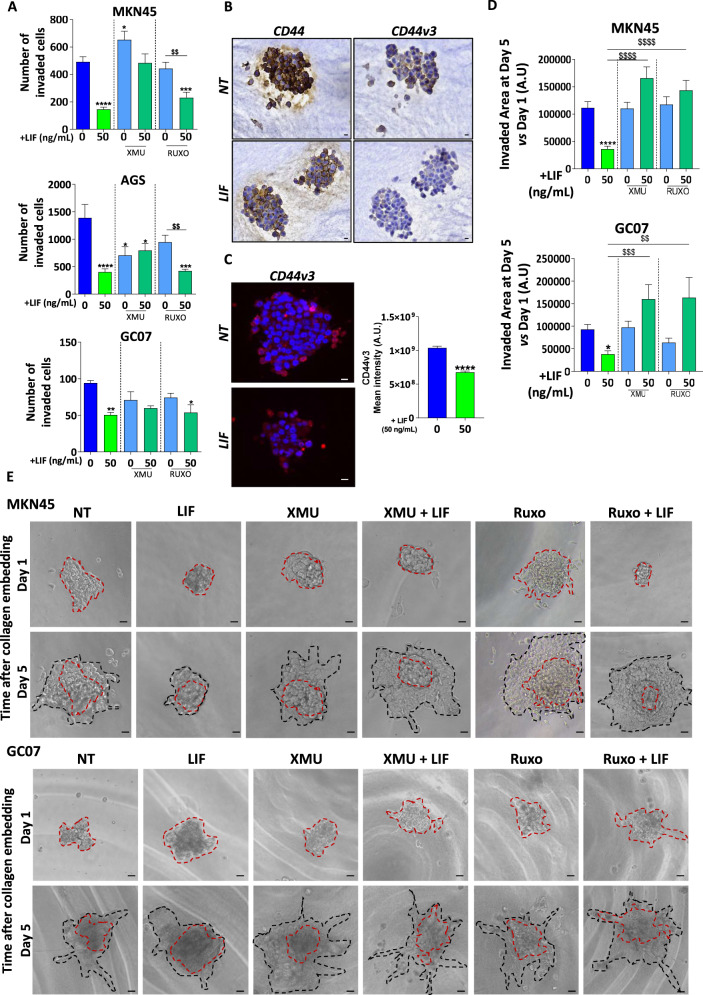
Leukaemia inhibitory factor decreases invasion of gastric cancer cells and Metastasis-initiating CSC. **A** Quantification of the number of invaded cells following the different treatments of GC cell lines and PDX cells. **B** Representative immunohistochemistry images of 3D collagen-invasion models stained with anti-CD44 and anti-CD44v3 antibodies. All cells were treated with 50 ng/mL LIF each 48 h. **C** Representative immunofluorescence images and quantification of collagen-embedded tumourspheres stained with anti-CD44v3 antibody. Tumourspheres were treated or not with LIF and CD44v3 Mean intensity was quantified. **D** and **E** Quantification and representative images of 3D collagen-invasion assay MKN45 GC cell line and GC07 GC PDX cells. Quantification was done for the invaded area on Day 5 (black dotted lines) vs. Day 1 (red dotted lines, treatments. All cells were treated with 50 ng/mL LIF (green) and/or 0.5 µM XMU-MP-1 (XMU) (emerald green) and 1 µM Ruxolitinib (emerald green) each 48 h. Inhibitors were added 30 min before LIF stimulation. Scale bars 20 µm, 4 < *n* < 5, *p < 0.05 and ****p < 0.0001 vs. untreated controls and $$*p* < 0.005, $$$*p* < 0.0005 and $$$$*p* < 0.0001 vs. the conditions indicated by the bars, all with ANOVA statistical analyses.

Furthermore, since LIF was found to inhibit the expression of certain invasion markers as well as CD44v3, expressed by MIC, functional 2D-invasion Boyden’s chamber assay was performed to validate this anti-invasion hypothesis (Fig. 3A). The number of cells invading type I collagen-coated microporous culture inserts decreased after LIF treatment, an effect blocked in LIFR-KD GC cells (Supplementary Fig. S4A), confirming the anti-invasive effect of LIF/LIFR signalling in all GC cell lines and PDX cells analysed. The Hippo pathway and not the JAK/STAT pathway was involved in LIF-dependent anti-invasive effects in AGS and MKN45 cell lines while the effect in GC07 PDX cells seemed to depend on both signalling pathways.

LIF consequently inhibits GC cell’s invasive properties through a decrease in EMT and invasion processes in vitro.

### LIF inhibits gastric CSC invasive properties

To further investigate whether LIF could affect the invasive capacity of CD44v3+ gastric MIC in vitro, these were selected according to previously established protocols in non-adherent culture conditions allowing the survival of only CSC [4, 5, 7]. Type-I collagen was then added to embed the tumourspheres and allow cells to invade in a 3D collagen gel. The collagen-embedded spheres were verified for CD44 and CD44v3 expression which was detected in the non-treated spheres (Fig. 3B, C). Immunofluorescence staining showed that CD44v3 was more expressed by the peripheral invasive cells (Fig. 3C). Importantly, LIF treatment decreased both CD44 and CD44v3 expression in gastric tumourspheres (Fig. 3B, C), suggesting that LIF may impact both CSC and MIC.

Collagen invasion by CSC was measured after 1 and 5 days. LIF decreased collagen-invasion area in the MKN45 cell line, GC07 PDX cells (Fig. 3D, E) as well as in the AGS cell line (Supplementary Fig. S4B, C). This anti-CSC invasive effect was dependent on the Hippo and JAK/STAT pathways activation by LIF signalling in all GC cell lines analysed (Fig. 3B, C and Supplementary Fig. S4B, C).

LIF is thus able to decrease invasive properties of GC cells and more particularly that of CD44v3+ invasive gastric CSC that may correspond to MIC.

### LIF inhibits EMT markers’ expression in invasive CSC

**Fig. 4:**
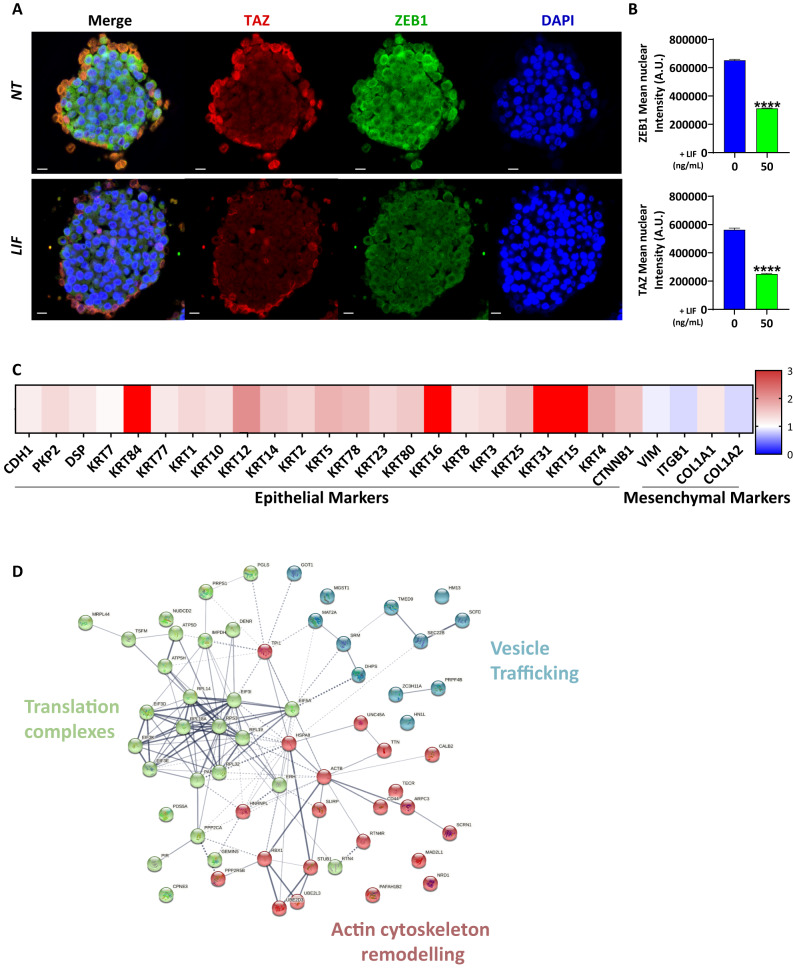
Leukaemia inhibitory factor decreases EMT markers in invasive gastric CSCs. **A** Representative immunofluorescent images of 3D collagen-invasion models of MKN45 GC cells stained with TAZ (red), ZEB1 (green) and DAPI (blue). **B** Relative quantification of cells with ZEB1 or TAZ-positive nucleus. Values represent Mean Nuclear intensity ± SEM, *n* = 3. **C** Relative protein expression profile of invasive fronts of LIF-treated spheres *vs*. non-treated spheres, following laser microdissection and LC–MS/MS mass spectrometry. Epithelial and mesenchymal markers are represented. **D** String software analysis of protein expression profile of invasive fronts of LIF-treated spheres vs. non-treated spheres. Scale bars 20 µm, ****p < 0.0001 vs. untreated controls, Mann–Whitney statistical analyses.

We then evaluated more specifically LIF’s anti-invasive properties on MIC within the CSC population in GC cell lines. LIF-induced decrease of invasion was related to a decrease in ZEB1 nuclear expression in the tumourspheres (Fig. 4A, B). Furthermore, the nuclear expression of the Hippo pathway oncogenic effector TAZ also decreased after LIF treatment, especially at the invasive periphery of the tumourspheres were localised CD44v3+ invasive cells in controls (Figs. 4A, B, 3C). Hippo effectors YAP and TAZ were found to be associated with ZEB1 in various cancers [27] and CSC invasive properties in GC [11]. LIF inhibitory effect on GC CSC invasive properties could be related to both ZEB1 and TAZ downregulation observed here (Fig. 4A, B).

Furthermore, these 3D-invasion models of gastric CSC were analysed by LC–MS/MS mass spectrometry proteomics analysis [28] coupled with laser microdissection to explore the proteome composition of CD44v3+ invasive edges of spheres treated or not with LIF. This showed that edges of LIF-treated spheres had a differential expression of EMT-related proteins with an increase in epithelial ones and a decrease in mesenchymal ones (Fig. 4C), reflecting EMT inhibition. Moreover, among the enriched pool of differentially expressed proteins were some involved in the translation of migration proteins, vesicle trafficking and actin cytoskeleton remodelling for migration and invasion (Fig. 4D). These were found to decrease after LIF treatment and could be related to the anti-invasive effect and anti-EMT phenotype observed. The expression of genes encoding these proteins was also found to decrease for most in the transcriptomic analysis of LIF-treated cells (Supplementary Fig. S1B).

### LIF impairs metastatic properties of MIC in vivo

**Fig. 5:**
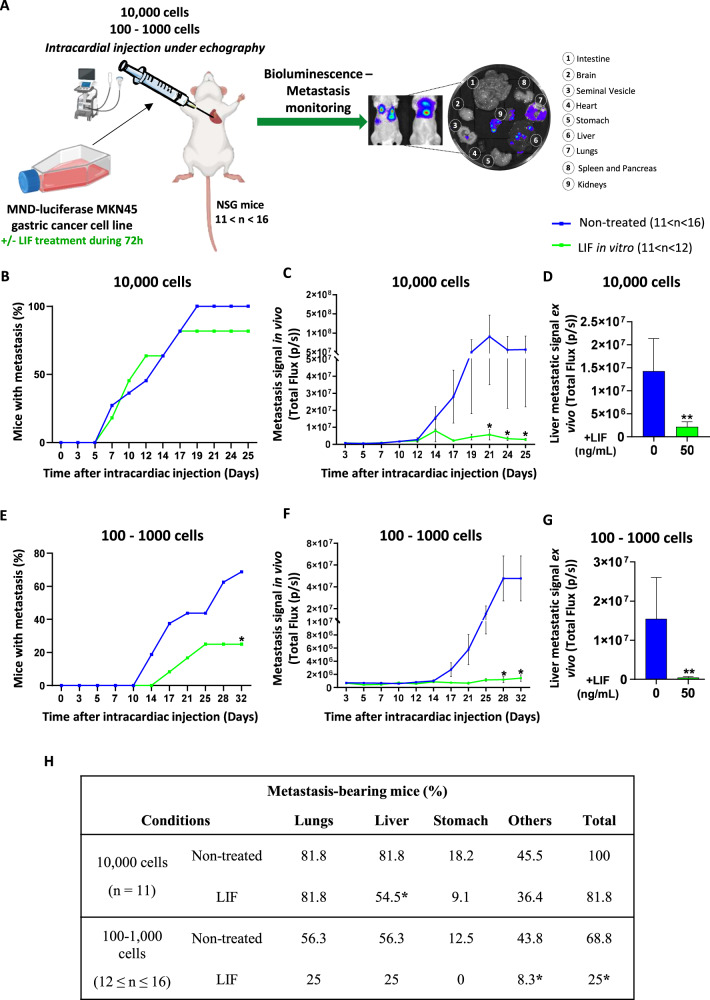
Leukaemia Inhibitory Factor presents anti-metastatic properties in vivo and affects organ colonisation. **A** Schematical representation of the in vivo experimental procedure and organs collected. Mice were injected with LIF-treated (green) or not (blue) MKN45 cells (10,000, 100 and 1000). Results obtained from 100 to 1000 cell conditions were grouped and named 100–1000. Metastasis follow-up of **B** and **E** Percentage of mice developing metastases; **C** and **F** Metastasis signal quantification in vivo; **D**, **G)** Liver metastatic signal quantification. Percentages were converted to binary values (0 = negative signal and 1 = positive signal) for statistical analysis. Total flux of bioluminescent signal ± SEM is represented in **C**, **D**, **F**, **G**. **H** Percentage of metastasis-bearing mice. 11–16 mice were injected with 100 to 10,000 MKN45-luciferase expressing cells previously treated or not with LIF, and bioluminescence was recorded to detect metastases in the different organs. *p < 0.05 and **p < 0.005 vs. untreated controls, Mann–Whitney statistical analyses.

LIF anti-invasive effect was finally evaluated in vivo in mice models of xenograft by intracardiac injection [29], reproducing cancer cell dissemination in the bloodstream and implantation process in secondary organs (Fig. 5A). Experiments were performed in limiting dilution with xenograft of either 10,000, 1000 or 100 luciferase-expressing MKN45 cells. When MKN45 cells were treated with LIF, they disseminated and colonised organs at a lesser extent than non-treated cells (Fig. 5B, E).

Mice metastatic follow-up showed that metastases were detected at day 5 for both conditions when 10,000 cells were injected, while a 4-day delay was noted for LIF-treated cells when 100–1000 cells were injected. In addition, at the endpoint, about 18.2% and 63.7% fewer mice developed metastases when 10,000 and 100–1000 LIF-treated cells were injected, respectively (Fig. 5B, E). Bioluminescence quantification of metastases revealed that overall metastasis formation was 96% and 97% lower in LIF-treated conditions showing that LIF decreased not only the number of mice having metastases but also the metastatic signal in positive mice (Fig. 5C, F).

Moreover, ex-vivo analysis of organs susceptible to carry metastases revealed that LIF decreased the appearance of liver metastasis (Fig. 5H) and when those were present, their signal was lower (Fig. 5D, G).

Accordingly, LIF decreases the metastatic potency of GC MIC in vivo.

### LIFR represents a novel prognostic marker for GC

**Fig. 6:**
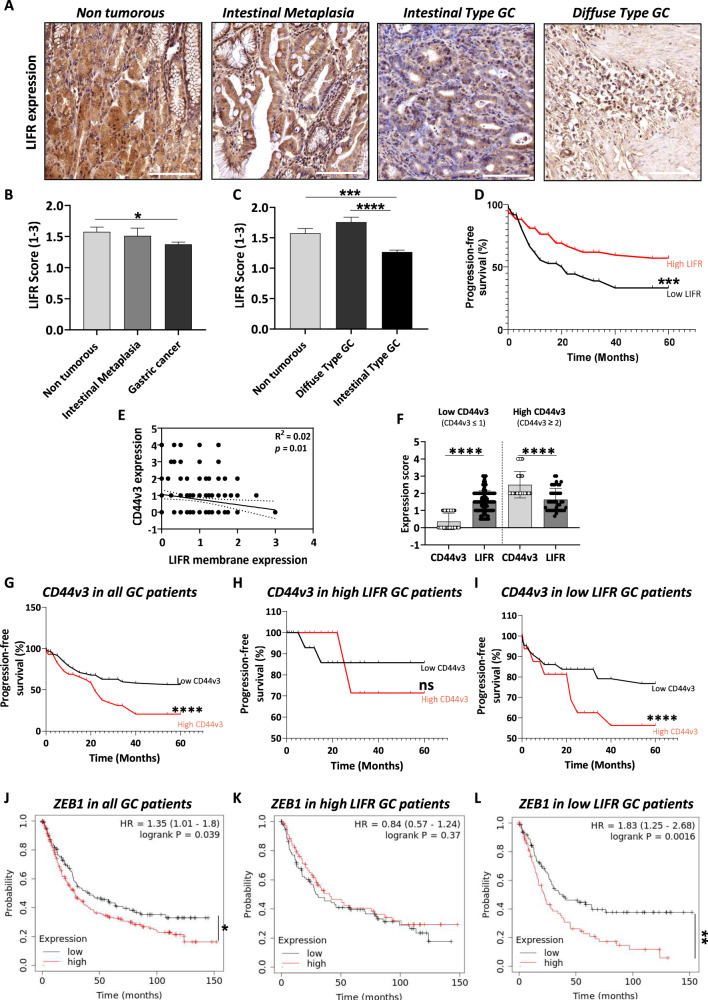
Leukaemia inhibitory factor receptor is downregulated in GC. **A** Representative images of the different types of TMA stained and analysed for LIFR expression (X20). Scale bars 100 µm. LIFR expression scored on TMA from GC patients. **B** and **C** Overall LIFR expression was analysed, and different comparisons were done: **B** Expression in Intestinal Metaplasia (*n* = 25) and GC (*n* = 146) were compared to non-tumorous tissue (*n* = 81), **C** GC was separated into the Laurèn classification-based subtypes diffuse (*n* = 33) and intestinal (*n* = 113) and compared to expression in non-tumorous tissue. Values represented mean LIFR scores according to the following criteria: 0: no expression, 1: 1–20%, 2: 20–50%, 3: >50. **D** Progression-free survival curves showing patients’ survival percentage according to overall LIFR low expression (LIFR ≤ 1, *n* = 40) and high expression (LIFR > 1, *n* = 68). **E** Correlation analysis of CD44v3 and LIFR-membrane expression scores (*n* = 133). **F** Expression scores of CD44v3 and LIFR in tumours having low CD44v3 (CD44v3 ≤ 1, *n* = 92) and high CD44v3 expression (CD44v3 ≥ 2, *n* = 42). **G–I** Progression-free survival curves showing survival percentage of GC patients (14 ≤ *n* ≤ 69) according to **G** CD44v3 expression in all patients independent of LIFR expression profile; **H** CD44v3 expression in patients having high LIFR expression; **I** CD44v3 expression in patients having low LIFR expression. Red bars represent high CD44v3 expression and black bars have low CD44v3 expression. **J–L** KMplot database analyses showing overall survival probability of GC patients (69 ≤ *n* ≤ 249) according to **J** ZEB1 expression in all patients independent of LIFR expression profile; **K** ZEB1 expression in patients having high LIFR expression; **L** ZEB1 expression in patients having low LIFR expression. Red bars represent high ZEB1 expression and black bars have low ZEB1 expression. *p < 0.05, **p < 0.005, ***p < 0.0005 and ****p < 0.0001 vs. the conditions indicated by the bars, ANOVA, Wilcoxon paired t-test statistical analyses, paired *t*-test and log-rank test.

Using TMA from a local collection of GC patients [8], we showed that LIFR is less expressed in GC compared to non-tumorous and preneoplastic intestinal metaplasia stage (Fig. 6A–C), especially in intestinal-type GC but not in diffuse-type GC according to the Laurèn classification of GC (Fig. 6B). Furthermore, LIFR low expression in tumours is related to GC patients’ poor 5-years progression-free survival (Fig. 6D), independent of GC histological subtypes. Interestingly, LIFR-expected membrane localisation was affected in GC and both in diffuse and intestinal-type GC (Supplementary Fig. S5A–C), while a nuclear expression was detected, and seemed mainly associated with diffuse-type GC. Nevertheless, though this difference in LIFR localisation was not related to the difference in patients’ prognosis (data not shown), LIFR membrane expression seemed to be inversely correlated to CD44v3 expression in the same patients (Fig. 6E). We previously reported the bad prognosis value of a high expression of CD44v3, the main marker of MIC in GC [8]. Interestingly, paired analysis of CD44v3 and LIFR expressions in tumours of GC patients showed that those having low CD44v3 expression had significantly higher LIFR expression than CD44v3, while high CD44v3-expressing tumours, shown to be of poor prognosis [8], had lower LIFR expression (Fig. 6F). Analysis of patients’ overall survival according to CD44v3 expression confirmed the bad prognosis of CD44v3 in GC (Fig. 6G). Nevertheless, when patients were grouped according to LIFR expression (Fig. 6H, I), CD44v3 high expression was of poor prognosis in low LIFR patients (Fig. 6I) while, in high LIFR patients, CD44v3 expression no longer distinguished survival, suggesting a protective effect of high LIFR expression.

Furthermore, in relation to the effect of LIF on ZEB1, GC patients’ overall survival according to ZEB1 was analysed on the KMplot database. High expression of ZEB1 is usually associated with low prognosis (Fig. 6J) but, when patients are grouped according to the mean expression of LIFR (Fig. 6K, L), in those with high LIFR expression, ZEB1 expression level is no longer related to patients’ prognosis (Fig. 6K) while in low LIFR expressing patients, ZEB1 high expression is of even worst prognosis (Fig. 6J compared to Fig. 6L).

These results thus demonstrate the prognosis value of high LIFR expression, also inversely correlated to the expression of the MIC marker CD44v3. This confirms the protective effect of high LIFR expression and signalling in GC, which may counteract metastatic properties and bad prognosis of CD44v3+ MIC and ZEB1+ mesenchymal type tumours in GC.

LIFR expression could thus constitute a novel prognosis marker in GC.

## Discussion

Despite LIF/LIFR signalling’s known pleiotropy [9, 21, 23, 30, 31], this pathway was found to negatively regulate GC cells’ proliferation [32] and tumorigenic properties [4]. Nevertheless, its effect on GC cells’ invasion and CSC metastatic properties remained poorly described.

This study aimed at deciphering the role of LIF in GC metastatic context, a particularly crucial aspect of gastric carcinogenesis leading to bad prognosis and in which GC patients are no longer eligible for surgery. Metastasis can be decomplexified in a series of steps, each important in this process, from tumour cells evasion from the primary tumour to reach distant organs, to homing and initiation of secondary tumours. We have previously reported that LIF has anti-tumorigenic effects [4] by acting on CSC in GC but a possible relationship between EMT and metastatic dissemination remains to be deciphered. Here, we demonstrate that LIF decreases EMT-TF’s expression, activity, the expression of mesenchymal markers as well as the mesenchymal-like phenotype of GC cells. ZEB1 and SNAIL are two important transcription factors in the EMT process, induced in various cancers, among which GC [11, 33–36], and associated with poor prognosis. The EMT process is found to be responsible for the emergence of cells with stem-like properties, CSC, largely responsible for tumour heterogeneity and aggressiveness [5, 7, 11, 37]. EMT is also responsible for the mesenchymal phenotype as well as invasive and metastatic properties of MIC in several cancers including GC [8, 38–40]. These MICs can be detected in tumour-invasive fronts and in circulating tumour cells (CTC) by the expression of CD44 splicing variants such as CD44v6 in breast and colorectal cancers [38–40] and CD44v3 in GC [8]. In this study, we show that LIF decreased CD44v3 expression and invasion markers including MMP, as well as GC cells invasive properties in vitro. In addition, the use of dox-inducible LIFR-KD GC cells which countered LIF effects confirmed that LIF-induced decrease in EMT markers, CD44v3 expression and invasion capacity of GC cells can be attributed to LIF/LIFR signalling.

LIF’s effect on GC cells does not imply that it acts on CSC. Indeed, CSCs are a small population of cells representing less than 3.5% of the whole GC cells’ population [4, 5, 7]. Here, we selected gastric CSC, by applying a 3D-culture protocol, before analysing their invasive properties in response to LIF. Interestingly, LIF did not only affect the invasion of GC cells but also decreased CD44v3+ invasive cells at the periphery of tumourspheres embedded in collagen gels. LIF also decreased ZEB1 EMT-TF and TAZ expression in these invasive CSCs. We have previously demonstrated that ZEB1 expression is associated with that of the Hippo oncogenic effector TAZ in GC. Hippo pathway dysregulation has been related to CSC tumorigenic properties [10–12] but also invasive ones through an effect of TAZ on EMT-TF [11]. In this context, we demonstrated ZEB1 and TAZ co-repression in invasive CD44v3+ CSC after LIF treatment, suggesting a possible role of activated Hippo kinases in the downregulation of TAZ and EMT-TF axis and, as a consequence, of CD44v3 expression [4, 8, 11].

Hippo pathway’s role in the LIF-induced effect was investigated using XMU Hippo kinase inhibitor [4]. Indeed, XMU blocked LIF inhibition of EMT and GC cells and CSC invasive properties, confirming the role of Hippo kinases in LIF-induced anti-invasive effects. Furthermore, contrary to what was observed for gastric tumorigenic properties [4] where the JAK/STAT pathway was not involved, it seems here to be partly involved in anti-EMT and anti-invasion properties of LIF in CSC.

LIF/LIFR signalling has been poorly studied in a GC metastatic context. Nevertheless, Bian et al. demonstrated LIF as inducing GC proliferation, migration and invasion through the LIFR-Hippo pathway, in opposition to our observations. They however demonstrated that LIF effects in their model were not reversed by LIFR shRNA-depletion showing that these cannot be attributed to LIF/LIFR signalling as in our work. These opposing conclusions might be explained by the difference in cell lines as well as the origin of patients’ samples used in the study. In our work, the effects on EMT pass mainly through the LIFR and partly through the regulation of Hippo and JAK/STAT pathways, as demonstrated by the LIFR depletion experiments and the use of Hippo and JAK/STAT pathways inhibitors. In accordance with our study, Xu et al. have demonstrated LIF’s anti-proliferating effects through a G1-phase arrest in AGS and MKN45 cell lines, confirming our results.

Our study also shows that LIF acts on the metastatic behaviour of GC cells in vivo. Indeed, treating cells with LIF decreased their metastasis initiation capacity, leading to fewer mice with metastasis and less metastasis signal. This intracardiac injection strategy [29] allows for bypass of the putative effect of LIF on primary tumour growth if cells were xenografted orthotopically, in order to study only the capacity of LIF-treated GC cells’ survival ability as circulating tumour cells (CTC) in mice bloodstream, their specific metastatic niche homing and colonising ability. Studies show that metastatic cells are pre-destined to reach certain organs and the liver is a preferential site in about 48% of GC cases [41]. Here, we show that LIF is able to decrease liver colonisation by GC CTC and thus decrease liver metastasis.

Using TMA from 177 GC patients [5, 7, 11], we have demonstrated that LIFR is less expressed in gastric tumours compared to non-tumorous gastric mucosa and that low LIFR expression was correlated to bad prognosis for patients, contrary to the observations of Bian et al. made from a different cohort and without distinction of histological subtypes of GC. Nevertheless, our present results are in accordance with data from other groups which we analysed previously using Oncomine and KMplot database [4]. Although we cannot exclude the fact that LIF might be acting through other pathways than LIFR, our work clearly shows a difference in LIFR prognostic value and in its expression profile depending on the histological subtypes of GC. LIFR expression in GC thus shows its interest as a biomarker of prognosis.

In addition, low LIFR-expressing patients have the worst prognosis when ZEB1 expression is high. This correlates with our results suggesting that LIF/LIFR signalling is able to inhibit ZEB1 activity and have a protective effect in ZEB1+ mesenchymal type GC cases. Similarly, GC cases with high CD44v3 expression, known to be associated with metastasis and poor prognosis [8], had lower LIFR expression while low CD44v3 patients, having better prognosis, had higher LIFR expression which was protective. LIFR expression was also protective in these MIC-rich patients since high LIFR expression counteracted their poor prognosis while in patients with low LIFR expressing tumours, CD44v3-high remained of poor prognosis.

*In fine*, this study unveils LIF treatment as a possibility for the targeting of invasive CSC/MIC to prevent gastric metastatic disease. Nonetheless, caution should be taken considering LIF’s use due to its highly pleiotropic aspects. Further studies aiming at specifically addressing LIF to its target cells could help bypass the possible off-targets and secondary effects. Nevertheless, the detection of LIFR on GC tumours remains an interesting clue to better estimate the prognosis of GC patients.

## Materials and methods

### Gastric cancer cell lines and patient-derived xenograft cell culture

MKN45 (RRID:CVCL_0434) and AGS (RRID:CVCL_0139) ATCC cells lines, authenticated using short tandem repeat (STR) profiling within the last three years and mycoplasma-free PCR tested, were cultured in RPMI 1640-Glutamax and DMEM F12-Glutamax media respectively, supplemented with 10% heat-inactivated foetal bovine serum (FBS) (all from Thermo Fisher Scientific, Villebon sur Yvette, France) and 50 µg/ml vancomycin (Invitrogen, Cergy-Pontoise, France) at 37 °C in a 5% CO_2_ humidified atmosphere. Patient-derived xenograft (PDX) cells GC07 cells [12, 42] were cultured in DMEM F12-Glutamax medium, supplemented with 10% FBS and 50 µg/ml vancomycin.

### Leukaemia inhibitory factor, Hippo and JAK/STAT inhibitor treatments

Recombinant human leukaemia inhibitory factor (LIF, PeproTech, Neuilly-Sur-Seine, France) reconstituted in PBS, was used at 50 ng/mL [4]. Adherent cells were submitted to serum starvation by medium change 2 h before treatments. LIF treatments were carried out for 48 h in most experiments except for cells prepared for in vivo assays which underwent 72 h treatment with a repeat 48 h after the first stimulation and cells for 3D-invasion assays in which LIF was added each 48 h. MST1/2 inhibitor XMU-MP-1 (Selleckchem, Euromedex, Souffelweyersheim, France) was diluted in DMSO and used at 0.5 µM. Ruxolitinib (Stemcell Technologies, Grenoble, France) JAK1 inhibitor was also diluted in DMSO before use at 1 µM. Inhibitors were added 30 min before LIF to ensure respective signalisation pathways blocking prior to LIF stimulation.

### Agilent microarray

AGS, MKN45 and GC07 cells (*n* = 1 for each cell line, *n* = 3 GC cell lines) were treated or not with LIF for 48 h for Agilent microarray transcriptomic analysis as previously described [4, 10, 12].

### 3D collagen invasion assay

AGS (500), MKN45 (100) and GC07 (1 000) cells were seeded in 96-well culture plates, previously coated with a 10% poly-2-hydroxyethyl methacrylate (Sigma-Aldrich, Saint-Quentin Fallavier, France) solution in 95% (v/v) ethanol and left to dry overnight at 50–60 °C to make them non-adherent. Cells were cultured in non-adherent CSC-selective conditions as described for tumoursphere assay [4] in DMEM F12-Glutamax medium, supplemented with 0.3% glucose, 1:100 N2-supplement (all from Thermo Fisher), 20 ng/mL human epithelial growth factor, 20 ng/mL basic fibroblast growth factor and 5 µg/mL insulin (all from Sigma-Aldrich) at 37 °C in a 5% CO_2_, humidified atmosphere. MKN45 tumourspheres were grown in a serum-free medium while AGS and GC07 cells needed 2% FBS supplementation to grow. After CSC selection and formation of 3-day-old spheres, LIF and inhibitor treatments were carried out and 100 µL of rat-tail type 1 collagen solution, at a final concentration of 1 mg/mL, was added the day-after to each well. Collagen was allowed to polymerise for 24 h and photos of the spheres were taken at different times (Days 1 and 5) with a camera-equipped inverted-light microscope using ×20 objective (Olympus, Rungis, France). The invasive area was measured using ImageJ 1.53f51 software (National Institutes of Health) [43] and calculated according to the following formula: (Day 5 sphere area−Day 1 sphere area).

### Immunofluorescence staining

For 2D experiments, 50,000 cells were seeded on rat-tail type-I collagen-coated (Corning, New York, USA) glass coverslips in 24-well plates and treated or not with LIF. Cells were fixed with 4% paraformaldehyde solution (Electron Microscopy Sciences, Hatfield, UK) in cytoskeletal buffer, as previously described [4, 10, 11].

Immunofluorescence on tissue sections of paraffin-embedded collagen spheres followed the same protocol apart from an antigen demasking step carried out in 96 °C-hot pH 6 citrate buffer for 1 h. Primary antibodies used were mouse anti-human CD44v3 (R&D systems clone 3G5, Bio-Techne, Noyal Chatillon sur Seiche, France), rabbit anti-ZEB1 (Bethyl Laboratories, Montgomery, USA), rabbit anti-SNAI1 (H-130) (Santa Cruz Biotechnology, Bergheimer, Germany), mouse anti-TAZ (BD Biosciences, Grenoble, France); all at 1:100 for 1h30, and secondary fluorophore-labelled antibodies were Alexa Fluor® 488 goat anti-rabbit, Alexa Fluor® 594 donkey anti-mouse (1:200), Alexa Fluor® 647-Phalloidin (1:400), Alexa Fluor® 405 donkey anti-rabbit (1:250), combined with 4’-6-diamino-phenyl-indol (DAPI, 50 mg/mL) or DiTO™-3 (5 µM) (AAT Bioquest), for 1 h (all from Thermo Fisher). Images were taken using an Eclipse 50i epi-fluorescence microscope (Nikon, Champigny sur Marne, France) with NIS-BR acquisition software and a ×40 (numerical aperture, 1.3) oil immersion objective. ZEB1 and SNAIL mean nuclear intensity (mean Gray-value) or integrated density (mean Gray-value related to nuclear size variation for AGS) measurement was carried out using ImageJ 1.53f51 software [4, 10, 11, 43].

### Xenograft experiments and metastasis follow-up by bioluminescence imaging

Animal manipulations were performed in accordance with European directives for the care and use of animals and after approval by the local Ethics Committee CEEA50 of Bordeaux (agreement #31293). Animal manipulations were performed on anaesthetised immunodeficient NOD/SCID/IL-2Rnull (NSG) mice (7–14 weeks old) using 2% isoflurane (Belmont, Nicholas Piramal Limited, London, UK). Metastasis generation was performed by intracardiac injection of 100, 1000 or 10,000 MKN45 pMND-Luc LIF-treated or not cells (suspended in 100 µL PBS) in the left ventricle with ultrasound guidance (Aixplorer, Supersonic Imagine, France) [29]. Bioluminescence imaging was performed 10 min after d-luciferin (2.9 mg; 100 μL PBS, Promega) intra-peritoneal injection using the Lumina LT imaging system (Perkin Elmer Inc., Boston, MA, USA) at the Vivoptic platform (Univ. Bordeaux, CNRS INSERM TBM-Core UAR 3427 US 005). Mice were monitored for up to 28 days. At the endpoint, mice were sacrificed, and organs were recovered and analysed by ex vivo bioluminescence to determine organs having GC cells’ implantation [29].

### Human tissue samples collection and ethical statements

Paraffin-embedded samples were obtained from 177 consenting GC patients from 1999 to 2010, and processed following the agreement of the Clinical Research Direction and the Tumour and Cell Bank of the Bordeaux University Hospital Centre (Haut-Leveque Hospital, Pessac, France) as previously reported [5, 7, 11] and the French Ministry of Research (DC-2008-412).

### Immunohistochemistry

Immunostaining was carried out on 3 µm-thick tissue sections obtained from formalin-fixed paraffin-embedded human tissues or sphere inclusions, following previously established protocols [5, 7, 11]. Primary antibodies used were rabbit anti-LIFR (Abcam, Cambridge, UK) 1:200, anti-CD44 (BD Biosciences) 1:100 and anti-CD44v3 (R&D systems, clone 3G5) 1:4000. Relative double-blinded scoring of positive cells percentage was carried out according to the following criteria and score ranges: 0: no expression, 1: 1–20%, 2: 20–50%, 3: >50%. Subcellular localisations were noted and classified as follows: Membrane, Cytoplasmic, Nuclear (nuclear and peri-nuclear). Low expression of LIFR was defined as a score ≤ 1 and high expression score > 1.

### KMplot in silico database analysis

KMplot database tool (www.kmplot.com) [44] was used to analyse the overall survival probability of GC patients (69 ≤ *n* ≤ 249) according to ZEB1 and LIFR expression, as previously reported [4].

### Statistical analysis

Results are represented as mean ± SEM of at least three independent experiments. Statistical tests were carried out using GraphPad Prism software version 8.0.2 (La Jolla, CA, USA). ANOVA with Bonferroni as a post hoc test or Kruskal–Wallis test with Dunn’s test as post hoc was performed for multiple comparisons and the Mann–Whitney test or Student *t*-test was used for two groups’ comparisons. Paired *t*-test was used to compare expression scores in the same tumours. Progression-free survival was compared using a Wilcoxon paired t-test.

More information about cell transduction, RNA extraction and RT-qPCR, Agilent microarray, invasion assay, sphere paraffin embedding, immunohistochemistry, animal manipulation and cells’ xenograft, patients’ sample and TMA generation, proteomics and KMplot analysis are provided in the supplementary materials section.

### Supplementary information

supplementary materials

supplementary figures

## Data Availability

The data that support the findings of this study are available on request from the corresponding author.

## References

[1] Sung H, Ferlay J, Siegel RL, Laversanne M, Soerjomataram I, Jemal A, Bray F. Global Cancer Statistics 2020: GLOBOCAN Estimates of Incidence and Mortality Worldwide for 36 Cancers in 185 Countries. CA Cancer J Clin. 2021;71:209–49. 10.3322/caac.21660. Epub 4 Feb 2021. 10.3322/caac.21660 33538338

[2] Knight WR, Allum WH (2019). Gastric tumours. Medicine.

[3] Carrasco-Garcia E, García-Puga M, Arevalo S, Matheu A. Towards precision medicine: linking genetic and cellular heterogeneity in gastric cancer. Ther Adv Med Oncol 2018;10. 10.1177/1758835918794628. 10.1177/1758835918794628 PMC6116075 30181784

[4] Seeneevassen L, Giraud J, Molina-Castro S, Sifré E, Tiffon C, Beauvoit C, et al. Leukaemia inhibitory factor (LIF) inhibits cancer stem cells tumorigenic properties through hippo kinases activation in gastric cancer. Cancers (Basel) 2020;12. 10.3390/cancers12082011. 10.3390/cancers12082011 PMC7464447 32707998

[5] Nguyen PH, Giraud J, Chambonnier L, Dubus P, Wittkop L, Belleannée G (2017). Characterization of biomarkers of tumorigenic and chemoresistant cancer stem cells in human gastric carcinoma. Clin Cancer Res.

[6] Nguyen PH, Giraud J, Staedel C, Chambonnier L, Dubus P, Chevret E (2016). All-trans retinoic acid targets gastric cancer stem cells and inhibits patient-derived gastric carcinoma tumor growth. Oncogene.

[7] Bessède E, Staedel C, Acuña Amador LA, Nguyen PH, Chambonnier L, Hatakeyama M (2014). Helicobacter pylori generates cells with cancer stem cell properties via epithelial–mesenchymal transition-like changes. Oncogene.

[8] Giraud J, Seeneevassen L, Rousseau B, Bouriez D, Sifré E, Giese A, et al. CD44v3 is a marker of invasive cancer stem cells driving metastasis in gastric carcinoma. Gastric Cancer 2022. 10.1007/s10120-022-01357-y. 10.1007/s10120-022-01357-y PMC9950191 36528833

[9] Seeneevassen L, Bessède E, Mégraud F, Lehours P, Dubus P, Varon C (2021). Gastric cancer: advances in carcinogenesis research and new therapeutic strategies. Int J Mol Sci.

[10] Molina-Castro SE, Tiffon C, Giraud J, Boeuf H, Sifre E, Giese A (2020). The Hippo kinase LATS2 controls *Helicobacter pylori*-induced epithelial–mesenchymal transition and intestinal metaplasia in gastric mucosa. Cell Mol Gastroenterol Hepatol.

[11] Tiffon C, Giraud J, Molina-Castro SE, Peru S, Seeneevassen L, Sifré E et al TAZ controls *Helicobacter pylori*-induced epithelial–mesenchymal transition and cancer stem cell-like invasive and tumorigenic properties. Cells 2020;9. 10.3390/cells9061462. 10.3390/cells9061462 PMC7348942 32545795

[12] Giraud J, Molina-Castro S, Seeneevassen L, Sifré E, Izotte J, Tiffon C et al. Verteporfin targeting YAP1/TAZ-TEAD transcriptional activity inhibits the tumorigenic properties of gastric cancer stem cells. Int J Cancer 2019. 10.1002/ijc.32667. 10.1002/ijc.32667 31489619

[13] Luo Q, Wang C, Jin G, Gu D, Wang N, Song J (2015). LIFR functions as a metastasis suppressor in hepatocellular carcinoma by negatively regulating phosphoinositide 3-kinase/AKT pathway. Carcinogenesis.

[14] Lei C, Lv S, Wang H, Liu C, Zhai Q, Wang S (2018). Leukemia inhibitory factor receptor suppresses the metastasis of clear cell renal cell carcinoma through negative regulation of the yes-associated protein. DNA Cell Biol.

[15] Chen D, Sun Y, Wei Y, Zhang P, Rezaeian AH, Teruya-Feldstein J (2012). LIFR is a breast cancer metastasis suppressor upstream of the Hippo-YAP pathway and a prognostic marker. Nat Med.

[16] Seeneevassen L, Martin OCB, Lehours P, Dubus P, Varon C Leukaemia inhibitory factor in gastric cancer: friend or foe? Gastric Cancer 2022. 10.1007/s10120-022-01278-w. 10.1007/s10120-022-01278-w 35106710

[17] Gearing DP, Gough NM, King JA, Hilton DJ, Nicola NA, Simpson RJ (1987). Molecular cloning and expression of cDNA encoding a murine myeloid leukaemia inhibitory factor (LIF). EMBO J.

[18] Smith AG, Nichols J, Robertson M, Rathjen PD (1992). Differentiation inhibiting activity (DIA/LIF) and mouse development. Dev Biol.

[19] Moreau JF, Donaldson DD, Bennett F, Witek-Giannotti J, Clark SC, Wong GG (1988). Leukaemia inhibitory factor is identical to the myeloid growth factor human interleukin for DA cells. Nature.

[20] Shi Y, Gao W, Lytle NK, Huang P, Yuan X, Dann AM (2019). Targeting LIF-mediated paracrine interaction for pancreatic cancer therapy and monitoring. Nature.

[21] Guo H, Cheng Y, Martinka M, McElwee K (2015). High LIFr expression stimulates melanoma cell migration and is associated with unfavorable prognosis in melanoma. Oncotarget.

[22] Buckley AM, Lynam-Lennon N, Kennedy SA, Dunne MR, Aird JJ, Foley EK (2018). Leukaemia inhibitory factor is associated with treatment resistance in oesophageal adenocarcinoma. Oncotarget.

[23] Gulluoglu S, Sahin M, Tuysuz EC, Yaltirik CK, Kuskucu A, Ozkan F, et al. Leukemia inhibitory factor promotes aggressiveness of chordoma. 2017. 10.3727/096504017X14874349473815. 10.3727/096504017X14874349473815 PMC7841199 28247842

[24] Harvey KF, Zhang X, Thomas DM (2013). The Hippo pathway and human cancer. Nat Rev Cancer.

[25] Chaffer CL, San Juan BP, Lim E, Weinberg RA (2016). EMT, cell plasticity and metastasis. Cancer Metastasis Rev.

[26] Dongre A, Weinberg RA (2019). New insights into the mechanisms of epithelial-mesenchymal transition and implications for cancer. Nat Rev Mol Cell Biol.

[27] Lehmann W, Mossmann D, Kleemann J, Mock K, Meisinger C, Brummer T (2016). ZEB1 turns into a transcriptional activator by interacting with YAP1 in aggressive cancer types. Nat Commun.

[28] Henriet E, Abou Hammoud A, Dupuy J-W, Dartigues B, Ezzoukry Z, Dugot-Senant N (2017). Argininosuccinate synthase 1 (ASS1): a marker of unclassified hepatocellular adenoma and high bleeding risk. Hepatology.

[29] Genevois C, Hocquelet A, Mazzocco C, Rustique E, Couillaud F, Grenier N (2017). In vivo imaging of prostate cancer tumors and metastasis using non-specific fluorescent nanoparticles in mice. Int J Mol Sci.

[30] Mathieu M-E, Saucourt C, Mournetas V, Gauthereau X, Thézé N, Praloran V (2012). LIF-dependent signaling: new pieces in the Lego. Stem Cell Rev Rep.

[31] Ma D, Jing X, Shen B, Liu X, Cheng X, Wang B (2016). Leukemia inhibitory factor receptor negatively regulates the metastasis of pancreatic cancer cells in vitro and in vivo. Oncol Rep.

[32] Xu G, Wang H, Li W, Xue Z, Luo Q (2019). Leukemia inhibitory factor inhibits the proliferation of gastric cancer by inducing G1-phase arrest. J Cell Physiol.

[33] Zhang G-J, Zhou T, Tian H-P, Liu Z-L, Xia S-S (2013). High expression of ZEB1 correlates with liver metastasis and poor prognosis in colorectal cancer. Oncol Lett.

[34] Murai T, Yamada S, Fuchs BC, Fujii T, Nakayama G, Sugimoto H (2014). Epithelial-to-mesenchymal transition predicts prognosis in clinical gastric cancer. J Surg Oncol.

[35] Okugawa Y, Toiyama Y, Tanaka K, Matsusita K, Fujikawa H, Saigusa S (2012). Clinical significance of zinc finger E-box binding homeobox 1 (ZEB1) in human gastric cancer. J Surg Oncol.

[36] Chen H, Lu W, Huang C, Ding K, Xia D, Wu Y (2017). Prognostic significance of ZEB1 and ZEB2 in digestive cancers: a cohort-based analysis and secondary analysis. Oncotarget.

[37] Mani SA, Guo W, Liao M-J, Eaton EN, Ayyanan A, Zhou AY (2008). The epithelial-mesenchymal transition generates cells with properties of stem cells. Cell.

[38] Todaro M, Gaggianesi M, Catalano V, Benfante A, Iovino F, Biffoni M (2014). CD44v6 Is a marker of constitutive and reprogrammed cancer stem cells driving colon cancer metastasis. Cell Stem Cell.

[39] Grillet F, Bayet E, Villeronce O, Zappia L, Lagerqvist EL, Lunke S (2017). Circulating tumour cells from patients with colorectal cancer have cancer stem cell hallmarks in ex vivo culture. Gut.

[40] Liu S, Cong Y, Wang D, Sun Y, Deng L, Liu Y (2014). Breast cancer stem cells transition between epithelial and mesenchymal states reflective of their normal counterparts. Stem Cell Rep.

[41] Riihimäki M, Hemminki A, Sundquist K, Sundquist J, Hemminki K (2016). Metastatic spread in patients with gastric cancer. Oncotarget.

[42] Giraud J, Bouriez D, Seeneevassen L, Rousseau B, Sifré E, Giese A, et al. Orthotopic patient-derived xenografts of gastric cancer to decipher drugs effects on cancer stem cells and metastatic dissemination. Cancers (Basel) 2019;11. 10.3390/cancers11040560. 10.3390/cancers11040560 PMC6520896 31010193

[43] Schindelin J, Arganda-Carreras I, Frise E, Kaynig V, Longair M, Pietzsch T (2012). Fiji: an open-source platform for biological-image analysis. Nat Methods.

[44] Lánczky A, Győrffy B (2021). Web-based survival analysis tool tailored for medical research (KMplot): development and implementation. J Med Internet Res.

