# Supplementary material for: Targeting metastasis-initiating cancer stem cells in gastric cancer with leukaemia inhibitory factor
Source: Cell Death Discov. 2024 Mar 7;10:120. doi: 10.1038/s41420-024-01839-1 (PMC10920825; doi:10.1038/s41420-024-01839-1)
Supplement: Supplementary file 1 — supplementary materials [file 41420_2024_1839_MOESM1_ESM.docx]

**Supplementary materials**

**Supplementary methods**

### Production of lentiviral particles and cell transduction

The lentiviral vectors used for the luciferase gene and LIFR shRNA transfer in cells were pRRLsin-MND-Luc-IRES2-ZsGreen-WPRE and pTRIPZ-shLIFR, respectively, produced by the Bordeaux University Vectorology platform, as previously described (PDOX). MKN45 GC cell line was transduced with a multiplicity of infection (MOI) of 30 lentiviral particles per cell for luciferase lentiviral vector and MKN45, AGS, and GC07 cells were transduced with lentiviral particles at a MOI of 10 for 24 h in RPMI 1640-Glutamax medium, supplemented with 10 mM Hepes (ThermoFisher Scientific, Villebon sur Yvette, France) for MKN45 and DMEM F12-Glutamax medium for AGS and GC07. After a 6-hour incubation period, medium containing 10% heat-inactivated FBS and 50 µg/ml vancomycin was added to the cultures. Medium was changed 12 h later and cells were subjected to selection by 1 µg/mL of puromycin before being amplified to be used for the experiments. Transduced cells with luciferase vector and pTRIPZ-shLIFR will be named pMND-Luc MKN45 cells and LIFR-KD cells, respectively hereafter. Luciferase vector transfection efficiency was verified by flow cytometry with about 100% cells ZsGreen-positive transduced cells. shLIFR expression was achieved by induction of LIFR-KD cells with 0.2 ng/mL of doxycycline (dox) for 48 h and transfection efficiency was evaluated by RFP fluorescence.

### RNA extraction and RTqPCR

100,000 MKN45 and AGS GC cell lines were plated in 12-well plates, serum-deprived the day-after and then treated or not with 50 ng/mL LIF for 48 h. Total RNA was extracted using TRIzol^TM^ reagent (ThermoFisher) according to manufacturer’s recommendations. Reverse transcription was then carried out with the Quantitech Reverse Transcription kit (QIagen, Netherlands) following manufacturer’s protocol. Real-time PCR was performed using SYBR-qPCR-Premix Ex-Taq (Takara, Shiga, Japan) and 0.5 µM specific primers (Supplemantary Table S1), as previously described ^1,2^. Analysis of amplified RNA samples was carried out using the 2^−∆∆CT^ method with TBP and HPRT1 as normalisers.

### Agilent microarray

RNAs extraction was carried out using RNeasy microkit (all from Qiagen, Netherlands) and RNA quantification was carried out using the TapeStation system (Agilent Technologies, Santa Clara, United States) to determine RNA integrity numbers (RIN). Agilent Sureprint G3 Human microarrays (8x60K, design 072363) were used for gene expression profiling at GeT‐TRiX facility (GenoToul, Génopole Toulouse Midi-Pyrénées, France), following manufacturer's instructions. One-Color Quick Amp Labelling kit (Agilent) and Agencourt RNAClean XP (Agencourt Bioscience Corporation, Beverly, MA, USA) were used to prepare cyanine-3 (Cy3) labelled cRNA from 25 ng of total RNA of each sample, according to manufacturer’s conditions. Dye incorporation and cRNA yield verification was done using Dropsense™ 96 UV/VIS droplet reader (Trinean, Ghent, Belgium). Cy3-labelled cRNA (600 ng) were hybridized on microarray slides following manufacturer’s instructions. Slides were washed and scanned immediately using Agilent G2505C Microarray Scanner and Agilent Scan Control A.8.5.1 software. Agilent Feature Extraction software v10.10.1.1 was used with default parameters for fluorescence signal extraction and only the most expressed probes values were represented. T-test statistical analysis was carried out to compare differentially expressed genes.

### Flow cytometry

AGS, MKN45 and GC07 cells were plated (1,000,000 cells) in T75 flasks, serum-deprived the day after and treated or not with 50 ng/mL LIF for 48 h. Cells were incubated with 1:20 mouse anti-human LIFR antibody (6G8 clone, given by Vincent Pitard, Immunoconcept CNRS UMR5164 Univ. Bordeaux, France) or isotype controls in ice-cold buffer containing PBS-0.5% bovine serum albumin (BSA, Gibco, ThermoFisher Scientific, Villebon sur Yvette, France) and 2 mM EDTA (Sigma-Aldrich, Saint-Quentin Fallavier, France) for 25 min at 4°C. Cells were washed with ice-cold buffer and incubated for 20 min with Alexa Fluor® 488 goat anti-mouse secondary antibodies (1:200) (ThermoFisher). Cells were washed thrice with ice-cold buffer and incubated for 10 min with 1:5000 SYTOX™ Blue. Cytometry acquisition was performed using a BD FACSCanto II instrument and the DIVA software (BD Biosciences, Le Pont de Claix, France). Dead cells were excluded based on side scatter characteristics and SYTOX™ Blue positivity. Data analysis was carried out using FCS express software (BD Biosciences).

### 2-D collagen invasion assay

50, 000 AGS and MKN45 cell lines and GC07 PDX cells were seeded in 24-wells plates, serum deprived after 24 h and treated with LIF for 48 h. Cells were then trypsin-recovered and 30, 000 cells were seeded in 8-µm pored Transwell® inserts, previously coated with rat-tail type 1 collagen solution (all from Corning, New York, USA). Inserts were placed in 24-well culture plates containing medium supplemented with 5% FBS and containing 50 ng/mL LIF or not. After 18 h incubation at 37°C, the Transwell® inserts were ﬁxed with 4% PFA in cytoskeletal buffer. Cells from the upper part of the insert were discarded using cotton swab and the insert membrane was processed for DAPI staining (ThermoFisher) 10 min before 3 PBS washings ^3–5^. Invaded cells were counted on 5 different randomly chosen fields per insert, under ZOE^TM^ Fluorescent Cell Imager (Bio-Rad, Marnes-la-Coquette, France).

### Spheres paraffin embedding

3-days MKN45 spheres, formed in 6-well plates, were treated with LIF. The spheres were collected the day-after, low-speed centrifugated (500 rpm for 5 min) and mixed with a collagen solution containing 1 mg/mL rat-tail type 1 collagen solution, 8% NAHCO_3_ (Sigma-Aldrich, Saint-Quentin Fallavier, France) and sphere-specific medium before replating in 6-well plates previously coated with 1 mg/mL the same collagen solution as before. 3 wells per condition were prepared and 5 days after collagen embedding, collagen gels were recovered, each condition was pooled, rinsed with PBS before adding 2-3 drops of undiluted Indian Ink (BIC, Clichy, France) for 30 s. Collagen gels were rinsed to remove excess ink and fixed in a 30 min-4% PFA bath before transferring in 70% ethanol. Blocks were processed with classical tissue paraffin-embedding protocol ^1,6–8^ before further histological applications.

### Immunohistochemistry

Immunostaining was carried out on 3 µm-thick tissue sections obtained from formalin-fixed paraffin-embedded human tissues or sphere inclusions. Antigen demasking step was carried out in 96°C-hot pH6 citrate buffer for 1 h. Primary rabbit anti-LIFR (Abcam, Cambridge, UK) antibodies were incubated for 2 h at 1:200, and mouse anti-CD44 (BD Biosciences) antibodies were incubated for 30 minutes at 1:100, both at room temperature. Detection was made using secondary horseradish peroxidase (HRP)-labelled HRP Horse Anti-Rabbit IgG Polymer Detection Kit ImmPRESS® (Vector Laboratories, Burlingame, United States). Immunostaining was revealed after incubation in liquid diaminobenzidine (DAB) chromogen substrate (DAKO, Agilent Technologies, Santa Clara, United States). Counterstaining was performed with Mayer′s hemalum solution (Sigma Aldrich Chimie, Saint-Quentin-Fallavier, France) before tissue dehydrating and slide mounting with Eukitt mounting medium (VWR, Fontenay-sous-Bois, France).

### Animal manipulation, xenograft experiments and metastasis follow up by bioluminescence imaging

Immunodeficient NOD/SCID/IL-2Rnull (NSG) mice (7 to 14-weeks old) were reared at the university facilities and maintained under 12-hour dark/light cycles with water and food provided *ad libitum* as previously reported ^1,7,8^. For intracardiac injection and imaging, mice were shaved with clippers. Bioluminescence imaging was performed using the Lumina LT imaging system (Perkin Elmer Inc., Boston, MA, USA) at the Vivoptic platform (Univ. Bordeaux, CNRS INSERM TBM-Core UAR 3427 US 005). D-luciferin (2.9 mg; 100 μL PBS, Promega) was injected intra-peritoneally. Bioluminescence acquisition (1 min, 4 x 4 binning) and photographs (100 ms) were taken 10 min after substrate injection. Data were analysed with Living Image software (Perkin Elmer) and Photon quantification was performed by drawing a region of interest ^9^.

### Tissue microarray generation

Paraffin-embedded samples for the Tissue microarray (TMA) generation, were obtained from 177 consenting GC patients, aged from 28 – 91 years old, from 1999 to 2010. The TMA blocks were produced using the MiniCore® 3 Tissue Arrayer (Excilone, Elancourt France). Areas of interest ware selected and extracted as 1 mm-diameter cylindrical tissue core triplicates and re-embedded into recipient blocks at defined array coordinates. Spleen (no expression of epithelial markers) and duodenum (expression of epithelial and stem cells markers at the bottom of crypts) were used as controls (n=3 samples per bloc). The TMAs included GC of different grades and histological sub-types determined after reanalysis and classification (G. Belleannée, pathologist, Bordeaux University Hospital) according to the last WHO and Lauren’s classification guidelines of gastric tumours, as well as non-tumour gastric mucosa distant from tumour sites (limits of exeresis), which may be healthy or involving pre-neoplastic lesions such intestinal metaplasia and dysplasia. Clinico-biological data including 5-years overall survival were also collected.

### Laser microdissection and proteomics analysis by LC-MS/MS mass spectrometry

Microscopy-directed 1 mm^2^ laser microdissection was carried out using a PALM type 4 automated micro-dissector (Zeiss Microscopy, France) on 5 µm-thick tissue sections previously stained haematoxylin and eosin. Areas of interest were selected beforehand from the invaded regions of MKN45 paraffin-embedded spheres treated or not with LIF (n=3). Sample preparation as well as LC-MS/MS Fusion Lumos mass spectrometry (ThermoFisher) was carried out as previously described ^10,11^. The Mascot 2.5 algorithm with Proteome Discoverer 2.5 Software (Thermo Fisher Scientific Inc.) was used for protein identification. It was used in batch mode for search against the UniProt Homo sapiens database (78 139 entries, Reference Proteome Set, release 2021_03) from http://www.uniprot.org/ website. Two missed enzyme cleavages were allowed. Raw LC-MS/MS data were treated by Proline software for feature detection, alignment, and quantification ^12^. Protein identification was only accepted with at least 2 specific peptides with a pretty rank=1 and with a protein FDR value less than 1.0% calculated using the “decoy” option in Mascot. Protein abundances were normalized using the "median ratio" option of Proline web. We applied an inference of missing data (5% of background). A paired t-test was performed for each cutting area. Differentially expressed proteins with a p value<0.05 were considered significant. STRING *v11.5* database ^13^ was used to visualise possible interactions between proteins from proteomics analysis and to classify them according to their function.

### KMplot in silico database analysis

The following JetSet best probes, 239952_at (ZEB1) and 225575_at (LIFR) were used. GSE62254 dataset was excluded during analysis as suggested by the database to avoid biased results. Patients’ samples were separated according to high or low expressions by the software’s best cut-off value auto-setting. *P-*values were calculated by a log rank test.

**Supplementary figure legends.**

**Supplementary Figure S1.** Relative LIF-treated cells gene expression profiles showing: **A)** Hippo pathway signature (Hippo kinases, Partners, Effectors, YAP/TAZ-TEAD target genes), **B)** Genes implicated in migration, vesicle trafficking and actin cytoskeleton remodelling in MKN45 and AGS GC cell lines and GC07 PDX cells. Agilent microarray transcriptomic analysis was carried out on LIF-treated cells compared to non-treated cells. The fourth row represent the mean expression fold change in all the cells analysed.

**Supplementary Figure S2. A)** Representative images of MKN45 and AGS GC cell lines and GC07 PDX cells treated or not with dox to induce LIFR-KD and RFP reporter expression (red). Cells were stained with nuclear marker DiTO™-3 (blue). Evaluation of dox-inducible LIFR-KD in AGS, MKN45 and GC07 GC cells by assessing LIFR expression by: **B)** RTqPCR and **C)** Flow cytometry, after cells treatment (red) or not (pink) with dox. **D)** Mesenchymal and **E)** Epithelial markers, assessed by RTqPCR, after treatment of AGS and MKN45 cells with (emerald green) or without (blue) LIF. Values represent fold change *vs.* non-treated LIFR-KD cells, 3 < n < 4. *p < 0.05, **p < 0.005, ***p < 0.0005 and ****p < 0.0001 *vs.* untreated controls with ANOVA statistical analyses. All cells were treated with 0.2 ng/mL dox followed by 50 ng/mL LIF, each for 48 h.

**Supplementary Figure S3. A)** Representative immunofluorescence images of MKN45 and AGS GC cell lines and GC07 PDX cells stained with anti-ZEB1 or anti-SNAIL antibodies (green). All cells were marked with DiTO™-3 nuclear stain (blue). **B/C)** Relative quantification of cells with ZEB1 or SNAIL-positive nucleus. Values for nuclear expression represent the mean nuclear intensity (MKN45 and GC07) or the nuclear integrated density (AGS) ± S.E.M., 3 < n < 4. All cells were treated with 0.2 ng/mL dox and/or 50 ng/mL LIF (green or emerald green) 48 h each. Scale bars 10 µm, **p < 0.005, ***p < 0.0005 and ****p < 0.0001 *vs.* untreated controls and *$$$$*p < 0.0001 *vs.* the conditions indicated by the bars, all with ANOVA statistical analyses.

**Supplementary Figure S4. A)** Quantification of number of invaded cells following the different treatments of GC cell lines and PDX cells. Representative images of 3D collagen-invasion assay of AGS GC cell line. **B)** Quantification of invaded area at Day 5 *vs.* Day 1, treatments. All cells were treated with 0.2 ng/mL dox and/or 50 ng/mL LIF (green and emerald green) each 48 h. Scale bars 20 µm, ** p < 0.005 *vs.* untreated controls with ANOVA statistical analyses.

**Supplementary Figure S5.** LIFR expression scored on TMA from GC patients **A-B)**. Membrane and Nuclear expression were analysed, and different comparisons were done: **A)** Expression in Intestinal Metaplasia and GC were compared to non-tumorous tissue, **B)** GC were separated into the Laurèn classification-based subtypes diffuse and intestinal and compared to expression in non-tumorous tissue. Values represented mean LIFR scores according to the following criteria: 0: no expression, 1: 1-20%, 2: 20-50%, 3: > 50%. 100 < n < 165, *p < 0.05, **p < 0.005, ***p < 0.0005 and ****p < 0.0001 *vs.* the conditions indicated by the bars, ANOVA statistical analyses. **C)** Representative images of the different types of TMA analysed (x40). Scale bars 50 µm. Arrows represent different subcellular localisations: membrane (yellow) and nuclear (green).

**Supplementary tables.**

**Supplementary Table S1. List of primers used for RT-qPCR analysis.**

| \| Gene \| Forward Sequence (5'-3') \| Reverse Sequence (5'-3') \| \| --- \| --- \| --- \| \| CDX2 \| GACGTGAGCATGTACCCTAGC \| GCGTAGCCATTCCAGTCCT \| \| CD44V3 \| GCACTTCAGGAGGTTACATC \| CTGAGGTGTCTGTCTCTTTC \| \| COL1A1 \| GATTCCCTGGACCTAAAGGTGC \| AGCCTCTCCATCTTTGCCAGCA \| \| CTNNB1 \| CACAAGCAGAGTGCTGAAGGTG \| GATTCCTGAGAGTCCAAAGACAG \| \| HPRT1 \| TGGTCAGGCAGTATAATCCA \| GGTCCTTTTCACCAGCAAGCT \| \| ILK \| GGACATGACTGCCCGAATTAGC \| GCGTCTGTTTGTGTCTTCAGGC \| \| ITGB1 \| GGATTCTCCAGAAGGTGGTTTCG \| TGCCACCAAGTTTCCCATCTCC \| \| ITGB3 \| CATGGATTCCAGCAATGTCCTCC \| TTGAGGCAGGTGGCATTGAAGG \| \| ITGA5 \| GCCGATTCACATCGCTCTCAAC \| GTCTTCTCCACAGTCCAGCAAG \| \| IGGAP1 \| AAAACTGGGACCAACCAAAGTGT \| GATGTGGCCTTTGGGATTCA \| \| KRT7 \| GCAGCAATGCCCTGAGCT \| TGCGGTCCGGATGGAATA \| \| MMP3 \| CACTCACAGACCTGACTCGGTT \| AAGCAGGATCACAGTTGGCTGG \| \| MMP7 \| GAACGCTGGACGGATGGTA \| CAAGTTCATGAGTTGCAGCATACA \| \| MMP14 \| ACTGCCAAGCCACCCTAAGA \| CTGAGCAACGAAGACCCTCTCT \| \| MUC2 \| ACAACTACTCCTCTACCTCCA \| GTTGATCTCGTAGTTGAGGCA \| \| SNAI1 \| ACAATGTCTGAAAAGGGACTGTGA \| CAGACCAGAGCACCCCATT \| \| SOX9 \| CGGAGGAAGTCGGTGAAG \| CTGGGATTGCCCCGAGTGCT \| \| SPP1 \| CACTCCAGTTGTCCCCACAGTA \| TTGACCTCAGTCCATAAACCACAC \| \| TBP \| TGCACAGGAGCCAAGAGTGAA \| CACATCACAGCTCCCCACCA \| \| ZEB1 \| TCCCAACTTATGCCAGGCAC \| CAGGAACCACATTTGTCATAGTCAC \| \| ZO-1 \| GTCCAGAATCTCGGAAAAGTGCC \| CTTTCAGCGCACCATACCAACC \| \| LIFR \| GCAGAGCCTGAAAACCATGT \| CCCCTCCTACAGGGTCATTT \| |
| --- | --- | --- | --- | --- | --- | --- | --- | --- | --- | --- | --- | --- | --- | --- | --- | --- | --- | --- | --- | --- | --- | --- | --- | --- | --- | --- | --- | --- | --- | --- | --- | --- | --- | --- | --- | --- | --- | --- | --- | --- | --- | --- | --- | --- | --- | --- | --- | --- | --- | --- | --- | --- | --- | --- | --- | --- | --- | --- | --- | --- | --- | --- | --- | --- | --- | --- | --- | --- | --- |

**Supplementary references.**

1 Giraud J, Molina-Castro S, Seeneevassen L, Sifré E, Izotte J, Tiffon C *et al.* Verteporfin targeting YAP1/TAZ-TEAD transcriptional activity inhibits the tumorigenic properties of gastric cancer stem cells. *Int J Cancer* 2019. doi:10.1002/ijc.32667.

2 Seeneevassen L, Giraud J, Molina-Castro S, Sifré E, Tiffon C, Beauvoit C *et al.* Leukaemia Inhibitory Factor (LIF) Inhibits Cancer Stem Cells Tumorigenic Properties through Hippo Kinases Activation in Gastric Cancer. *Cancers (Basel)* 2020; **12**. doi:10.3390/cancers12082011.

3 Molina-Castro SE, Tiffon C, Giraud J, Boeuf H, Sifre E, Giese A *et al.* The Hippo Kinase LATS2 Controls Helicobacter pylori-Induced Epithelial-Mesenchymal Transition and Intestinal Metaplasia in Gastric Mucosa. *Cellular and Molecular Gastroenterology and Hepatology* 2020; **9**: 257–276.

4 Tiffon C, Giraud J, Molina-Castro SE, Peru S, Seeneevassen L, Sifré E *et al.* TAZ Controls Helicobacter pylori-Induced Epithelial-Mesenchymal Transition and Cancer Stem Cell-Like Invasive and Tumorigenic Properties. *Cells* 2020; **9**. doi:10.3390/cells9061462.

5 Bessède E, Staedel C, Acuña Amador LA, Nguyen PH, Chambonnier L, Hatakeyama M *et al.* Helicobacter pylori generates cells with cancer stem cell properties via epithelial-mesenchymal transition-like changes. *Oncogene* 2014; **33**: 4123–4131.

6 Giraud J, Bouriez D, Seeneevassen L, Rousseau B, Sifré E, Giese A *et al.* Orthotopic Patient-Derived Xenografts of Gastric Cancer to Decipher Drugs Effects on Cancer Stem Cells and Metastatic Dissemination. *Cancers (Basel)* 2019; **11**. doi:10.3390/cancers11040560.

7 Nguyen PH, Giraud J, Chambonnier L, Dubus P, Wittkop L, Belleannée G *et al.* Characterization of Biomarkers of Tumorigenic and Chemoresistant Cancer Stem Cells in Human Gastric Carcinoma. *Clin Cancer Res* 2017; **23**: 1586–1597.

8 Nguyen PH, Giraud J, Staedel C, Chambonnier L, Dubus P, Chevret E *et al.* All-trans retinoic acid targets gastric cancer stem cells and inhibits patient-derived gastric carcinoma tumor growth. *Oncogene* 2016; **35**: 5619–5628.

9 Genevois C, Hocquelet A, Mazzocco C, Rustique E, Couillaud F, Grenier N. In Vivo Imaging of Prostate Cancer Tumors and Metastasis Using Non-Specific Fluorescent Nanoparticles in Mice. *International Journal of Molecular Sciences* 2017; **18**: 2584.

10 Henriet E, Abou Hammoud A, Dupuy J-W, Dartigues B, Ezzoukry Z, Dugot-Senant N *et al.* Argininosuccinate synthase 1 (ASS1): A marker of unclassified hepatocellular adenoma and high bleeding risk. *Hepatology* 2017; **66**: 2016–2028.

11 Dourthe C, Julien C, Di Tommaso S, Dupuy J-W, Dugot-Senant N, Brochard A *et al.* Proteomic Profiling of Hepatocellular Adenomas Paves the Way to Diagnostic and Prognostic Approaches. *Hepatology* 2021; **74**: 1595–1610.

12 Bouyssié D, Hesse A-M, Mouton-Barbosa E, Rompais M, Macron C, Carapito C *et al.* Proline: an efficient and user-friendly software suite for large-scale proteomics. *Bioinformatics* 2020; **36**: 3148–3155.

13 Szklarczyk D, Gable AL, Nastou KC, Lyon D, Kirsch R, Pyysalo S *et al.* The STRING database in 2021: customizable protein-protein networks, and functional characterization of user-uploaded gene/measurement sets. *Nucleic Acids Res* 2021; **49**: D605–D612.
