# Supplementary material for: Targeting metastasis-initiating cancer stem cells in gastric cancer with leukaemia inhibitory factor
Source: Cell Death Discov. 2024 Mar 7;10:120. doi: 10.1038/s41420-024-01839-1 (PMC10920825; doi:10.1038/s41420-024-01839-1)

Supplementary Figure S1

A

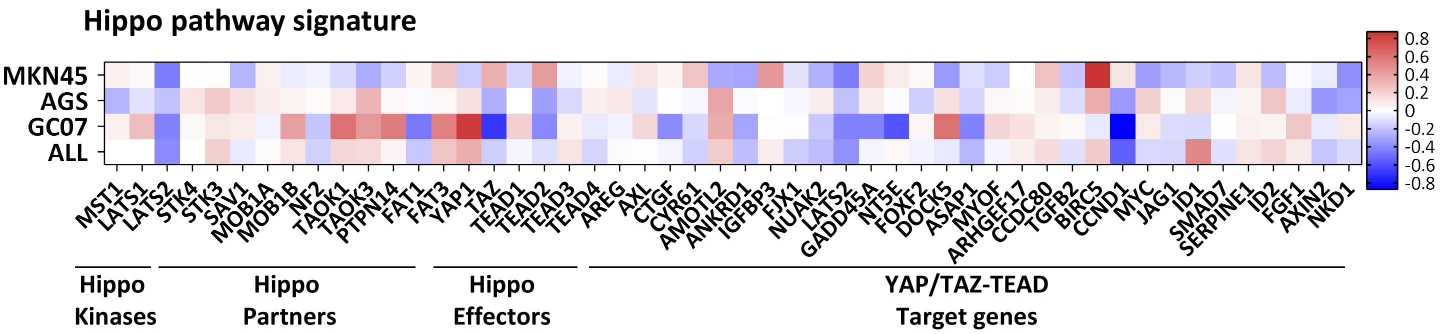

B

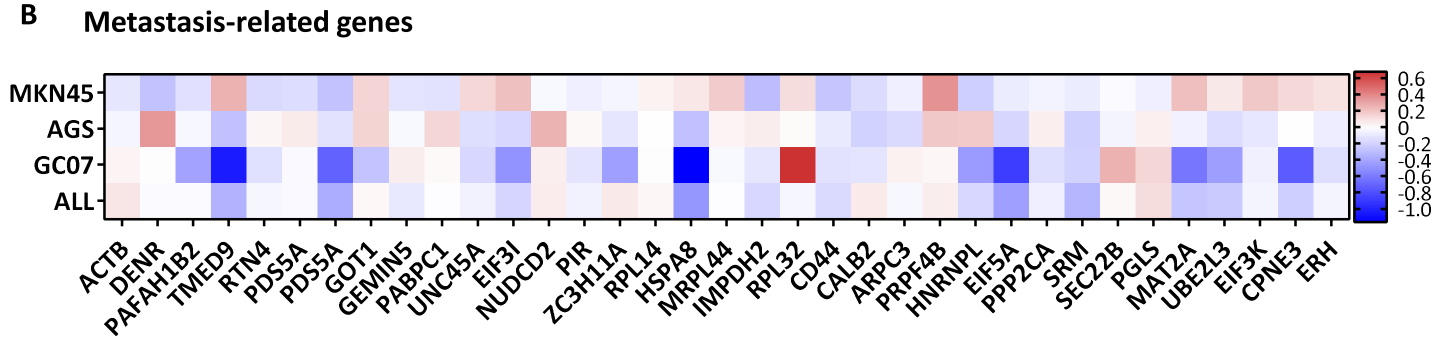

Supplementary Figure S2

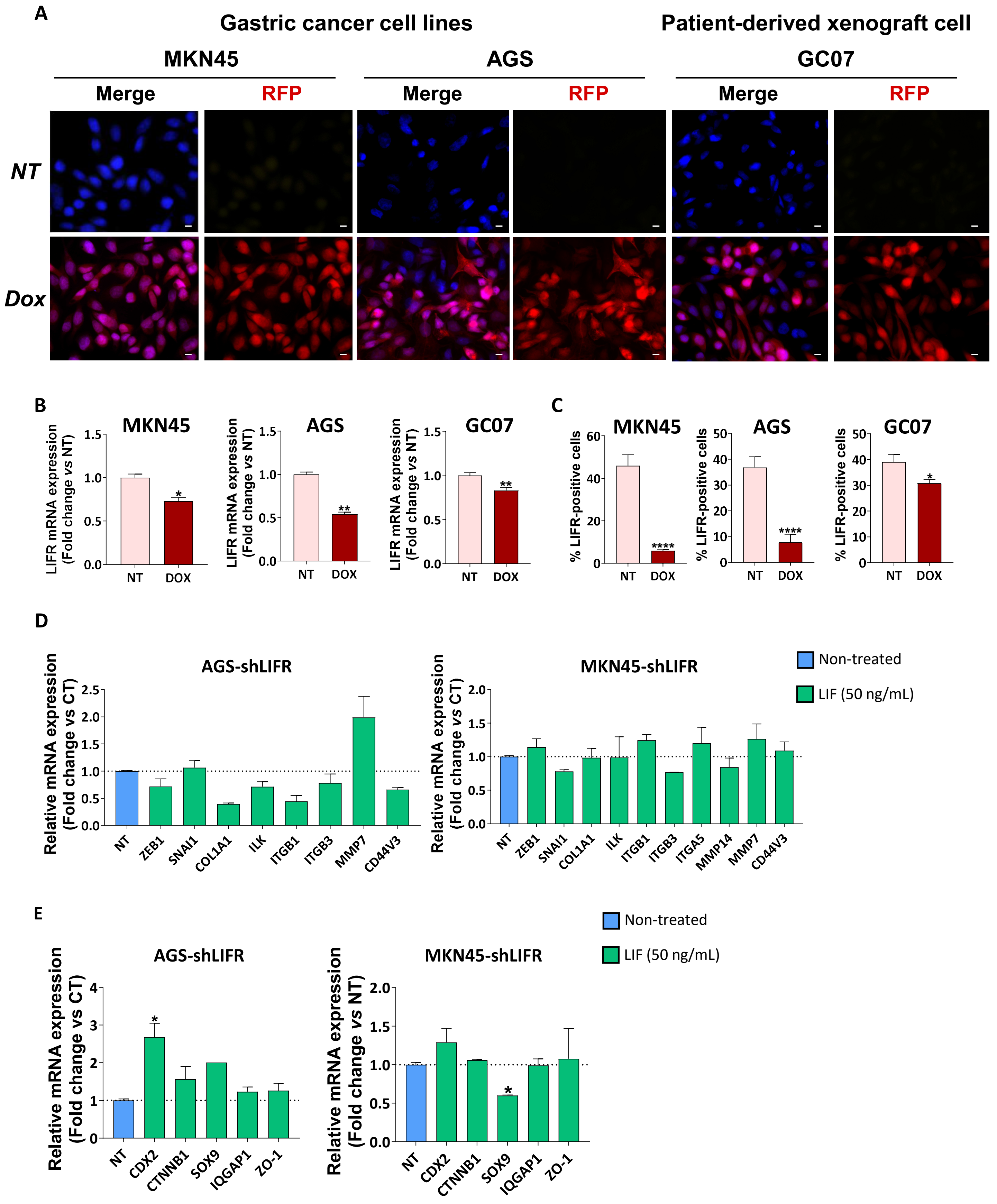

Supplementary Figure S3

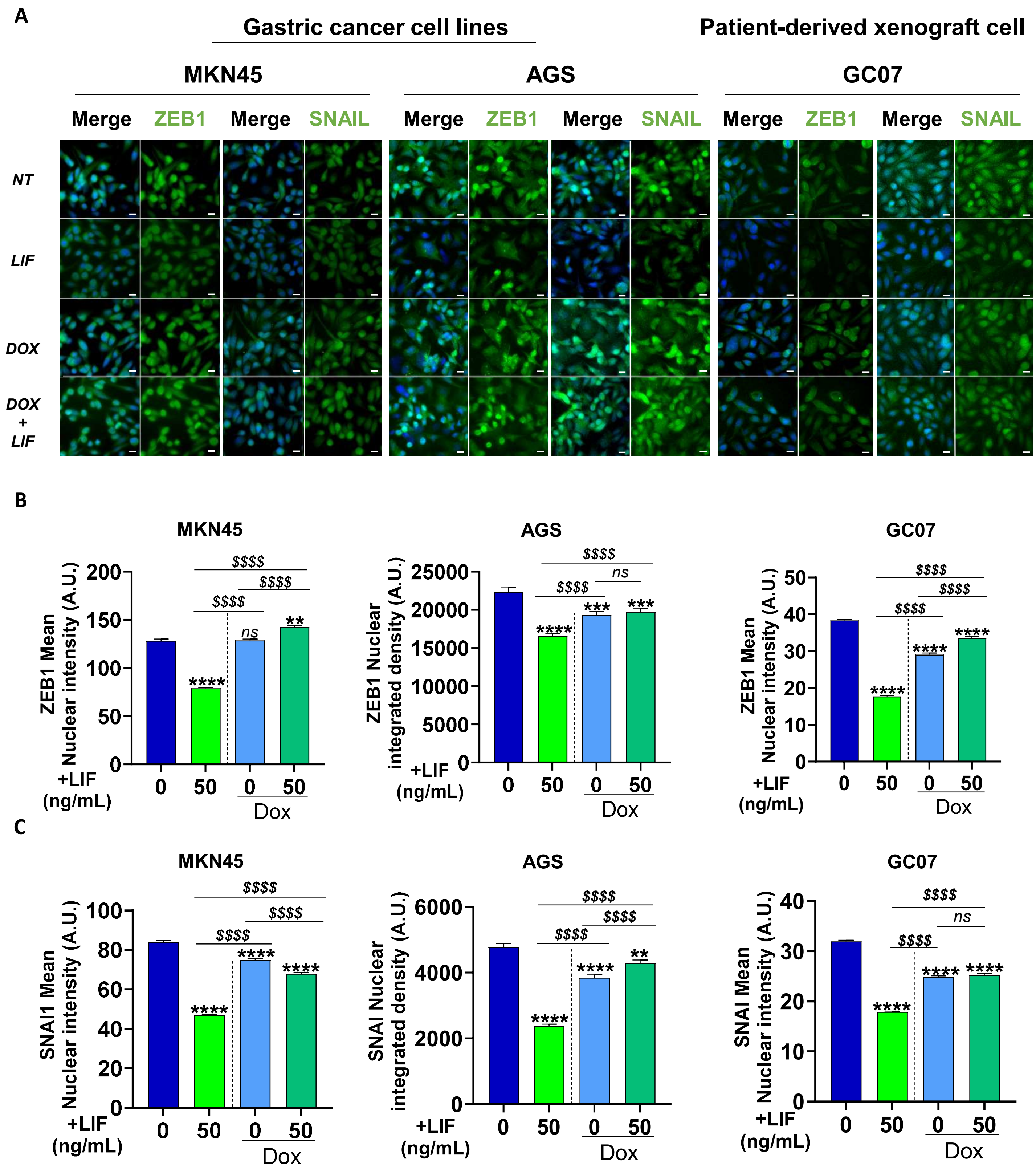

Supplementary Figure S4

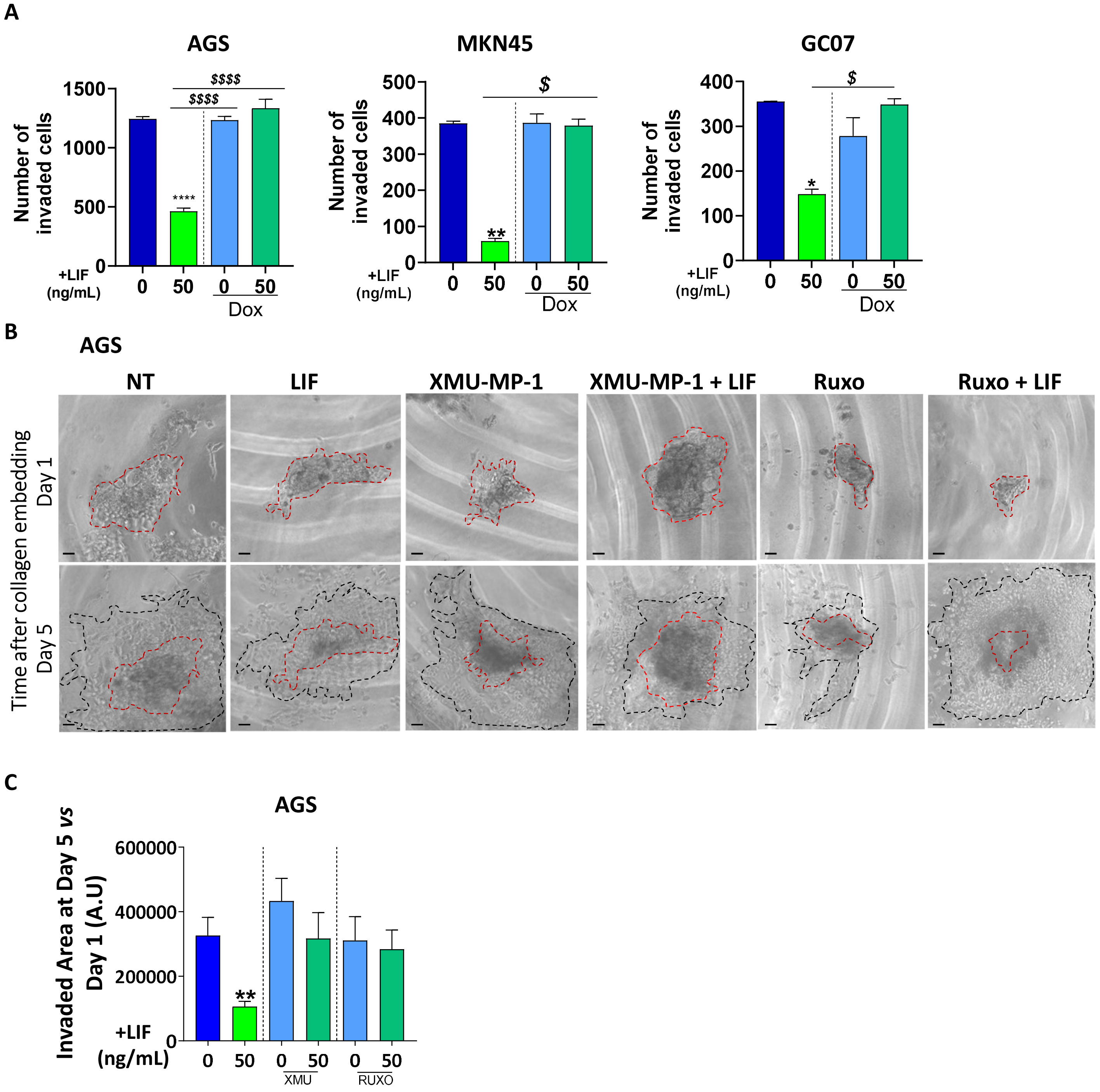

Supplementary Figure S5

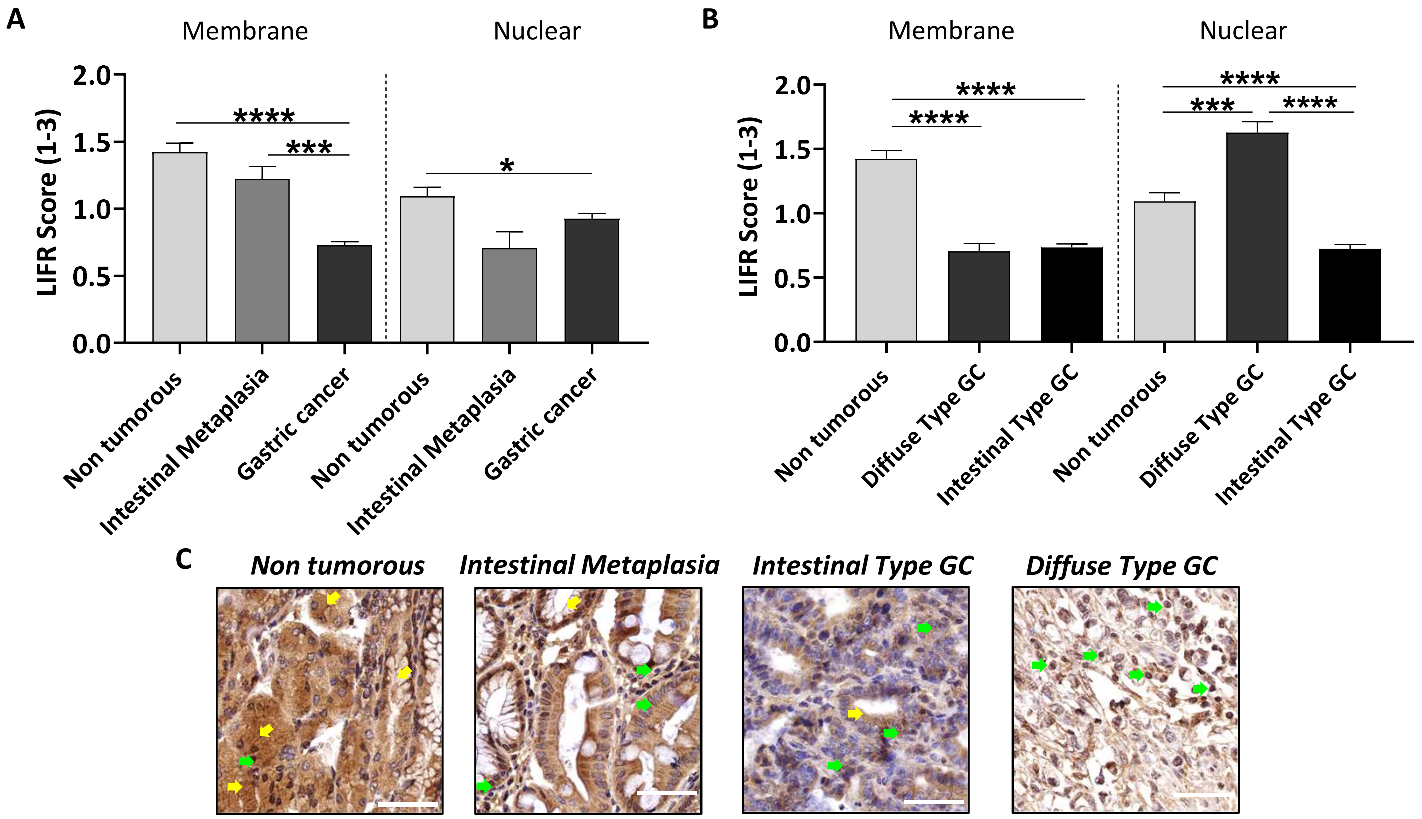

Supplement: Supplementary file 2 — supplementary figures [file 41420_2024_1839_MOESM2_ESM.pdf]
